# DANCE: a deep learning library and benchmark platform for single-cell analysis

**DOI:** 10.1186/s13059-024-03211-z

**Published:** 2024-03-19

**Authors:** Jiayuan Ding, Renming Liu, Hongzhi Wen, Wenzhuo Tang, Zhaoheng Li, Julian Venegas, Runze Su, Dylan Molho, Wei Jin, Yixin Wang, Qiaolin Lu, Lingxiao Li, Wangyang Zuo, Yi Chang, Yuying Xie, Jiliang Tang

**Affiliations:** 1 Department of Computer Science and Engineering, Michigan State University, East Lansing, USA; 2 Department of Computational Mathematics, Science and Engineering, Michigan State University, East Lansing, USA; 3 Department of Statistics and Probability, Michigan State University, East Lansing, USA; 4 Department of Biostatistics, University of Washington, Seattle, USA; 5 Department of Computer Science, Zhejiang University of Technology, Zhejiang, China; 6 Department of Bioengineering, Stanford University, Palo Alto, USA; 7 Department of Computer Science, Boston University, Boston, USA; 8 School of Artificial Intelligence, Jilin University, Jilin, China

**Keywords:** Deep learning, Benchmarking, Single-cell multimodal analysis, Single-cell spatial analysis, Gene imputation, Cell type annotation, Clustering, Multimodality integration, Spatial domain identification, Cell type deconvolution

## Abstract

DANCE is the first standard, generic, and extensible benchmark platform for accessing and evaluating computational methods across the spectrum of benchmark datasets for numerous single-cell analysis tasks. Currently, DANCE supports 3 modules and 8 popular tasks with 32 state-of-art methods on 21 benchmark datasets. People can easily reproduce the results of supported algorithms across major benchmark datasets via minimal efforts, such as using only one command line. In addition, DANCE provides an ecosystem of deep learning architectures and tools for researchers to facilitate their own model development. DANCE is an open-source Python package that welcomes all kinds of contributions.

**Supplementary Information:**

The online version contains supplementary material available at 10.1186/s13059-024-03211-z.

## Background

Single-cell profiling technology has undergone rapid development in recent years, spanning from single modality profiling (RNA, protein, and open chromatin) [1–9], multimodal profiling [10–14], to spatial transcriptomics [15–22]. The fast revolution in this field has encouraged an explosion in the number of computational methods, especially machine learning-based methods. However, the diversity and complexity of current methods make it difficult for researchers to reproduce the results as shown in the original papers. The major challenges include no publicly available codebase, hyperparameter tuning, and differences between programming languages. Furthermore, a systematic benchmarking procedure is necessary to comprehensively evaluate methods since the majority of existing works have only reported their performance on limited datasets and comparison with insufficient methods. Therefore, a generic and extensible benchmark platform with comprehensive benchmark datasets and metric evaluation is highly desired to easily reproduce any algorithm other than state-of-art methods under different tasks across popular benchmark datasets via minimal efforts (e.g., only one command line). Considering deep learning methods like graph neural networks (GNNs) [11, 23–28] have shown promising performance in single-cell analysis, the customized interfaces of such tools are largely missing in the existing packages. Those motivate the development of our DANCE system which not only acts as a benchmark platform but also provides customized deep learning infrastructure interfaces to help researchers conveniently develop their models.

In this work, we present DANCE as a deep learning library and benchmark platform to facilitate research and development for single-cell analysis. DANCE provides an end-to-end toolkit to facilitate single-cell analysis algorithm development and fair performance comparison on different benchmark datasets. DANCE currently supports 3 modules, 8 tasks, 32 models, and 21 datasets. One of the highlights of DANCE is the reproducibility of models. The diverse programming languages and backend frameworks of existing methods make systematic benchmark evaluation challenging for fair performance comparison. In such case, we implement all models in a unified development environment based on python language using Pytorch [29], Deep Graph Library (DGL) [30], and PyTorch Geometric (PyG) [31] as backbone frameworks. In addition, we formulate all baselines into a generic fit-predict-score paradigm. From the reproducibility perspective, for each task, every implemented algorithm is fine-tuned on all collected standard benchmarks via grid search to get the best model, and the corresponding hyperparameters are saved into only one command line for user reproducibility. We also provide one example for each model as a reference.

## Results and discussion

### Pipeline overview

**Fig. 1:**
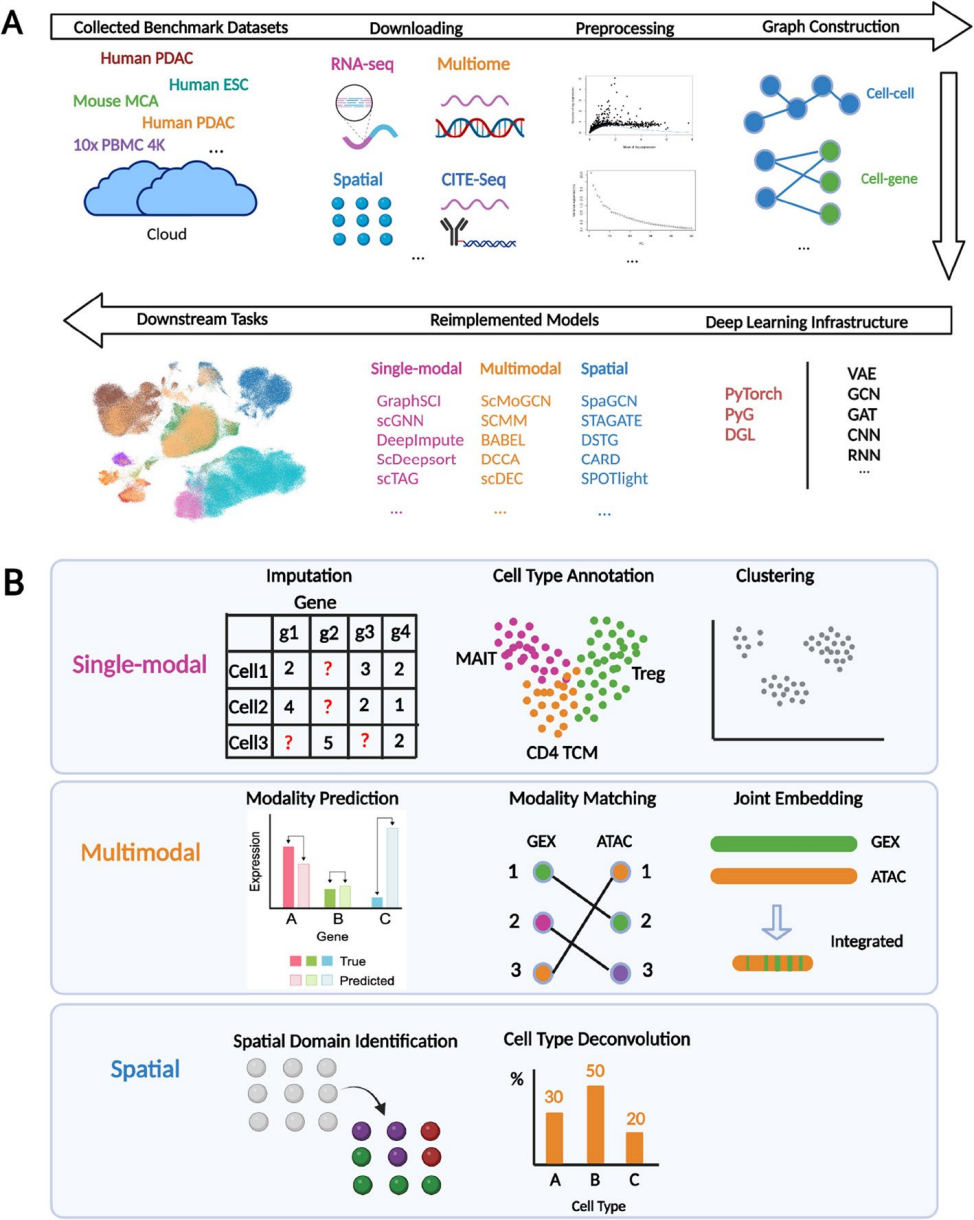
User perspective of DANCE platform. **a** Overview of single-cell omics analysis pipeline with DANCE platform. Benchmark datasets by the task are organized and cached on the cloud in advance for users’ usage. Those data cover scRNA-seq data, multimodal single-cell data like Chromium Single Cell Multiome ATAC + Gene Expression and cellular indexing of transcriptomes and epitopes (CITE-seq), and spatially resolved transcriptomic data. After automatic data downloading from the cloud, the DANCE built-in preprocessing and graph construction (required for graph neural networks model development) are then executed. Subsequently, users can build up their own models via customized deep learning model module in DANCE or utilize our reimplemented state-of-the-art deep learning models in DANCE to accomplish downstream tasks. **b** Currently supported downstream tasks in DANCE

Briefly, the single-cell analysis pipeline with DANCE platform includes data collection, data downloading, data processing (preprocessing and graph construction), and model development on specific downstream tasks (Fig. 1a).

#### Benchmark dataset collection

We first collect standard and popular benchmark datasets for each supported downstream task in DANCE. Then, those datasets are organized and cached by dataset name on the cloud.

#### Data downloading

For each task, DANCE provides a generic interface to load datasets. Since all benchmark datasets supported by DANCE are cached on the cloud in advance, users do not have to download their interested datasets manually. They just need to specify a dataset name when calling the data loader interface. For example, we can run graph-sc model on 10X PBMC dataset for the clustering task using the following command line:
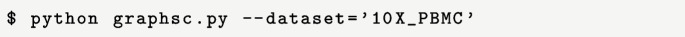


#### Data processing

After data loading, a collection of data processing methods are provided before model training. They are divided into two parts: preprocessing and graph construction.

- *Preprocessing*: We provide rich preprocessing functions such as normalization, dimension reduction, and gene filtering. Take graph-sc model as an example, we filter out the rarely expressed genes and normalize the remaining to obtain the same total count for each cell. Then, only the highly expressed genes (top 3000 by default) are kept for clustering [25].
- *Graph construction*: This is required for GNN-based method. Before model training, we have to convert data to graphs in preparation for graph operations. DANCE provides a variety of ways of graph construction. In graph-sc implementation, we construct a weighted heterogeneous cell-to-gene graph, where the types of nodes can be cell and gene nodes. There are weighted edges between cell nodes and the expressed gene nodes. Let the raw data cell-gene matrix be *X*, then the weight of gene *i* to cell *j* is $w_{ij}=\frac{X[i,j]}{\sum _{k=0}^m X[k,j]}$. There is no edge linked between any pairs of cell or gene nodes.

#### Model development

All types of deep learning models by task have been reimplemented in DANCE with a generic backend framework and unified interface for usage. Users can directly apply them to their interested downstream tasks or build up their own model via our provided customized deep learning module in DANCE.

As shown in Fig. 1b, DANCE presently supports tasks of data spanning through single modality profiling, multimodal profiling, and spatial transcriptomics, which correspond to three stages of single-cell technology development. For a single-modal module, only a single modality like gene expression in the cell can be obtained for analysis. Imputation, cell type annotation, and clustering tasks are supported under this module. For the multimodal module, multiple modalities for the cell can be accessed. For example, CITE-seq can provide both gene expression and protein data for analysis. Modality prediction, modality matching, and joint embedding are currently supported. For the spatial transcriptomics module, the spatial location of the cell in the tissue can be obtained additionally. Spatial domain identification and cell type deconvolution are presently placed under this module. For more details about each task, please refer to the “Methods” section.

### Deep learning library

We have seen the rapid development of deep learning in single-cell analysis in recent years [32–39] due to its capability of handling huge, high-dimensional, and sparse data. Among them, GNN, as a branch of deep learning, is playing an increasingly important role in the filed of single-cell analysis [25, 26, 40–44] because it is natural to represent cell-gene in a graph, include prior knowledge into graphs, and extract gene-gene patterns hidden from cell-gene relations via propagation. To facilitate the development of deep learning models in this field, we not only provide all kinds of basic deep learning model implementations like commonly used autoencoders (AEs) [45], generative adversarial networks (GANs) [46], and convolutional neural network (CNN) [47, 48] but also support all types of graph operations like graph convolutional network (GCN) [49] and graph attention network (GAT) [50]. What is more, due to the fact that the original single-cell data is not a graph, we also design several interfaces for users to construct various graphs, like cell-cell, cell-gene, and gene-gene graphs after which one of the GNNs is applied.

### Benchmark overview: modules, tasks, models and benchmark datasets

**Fig. 2:**
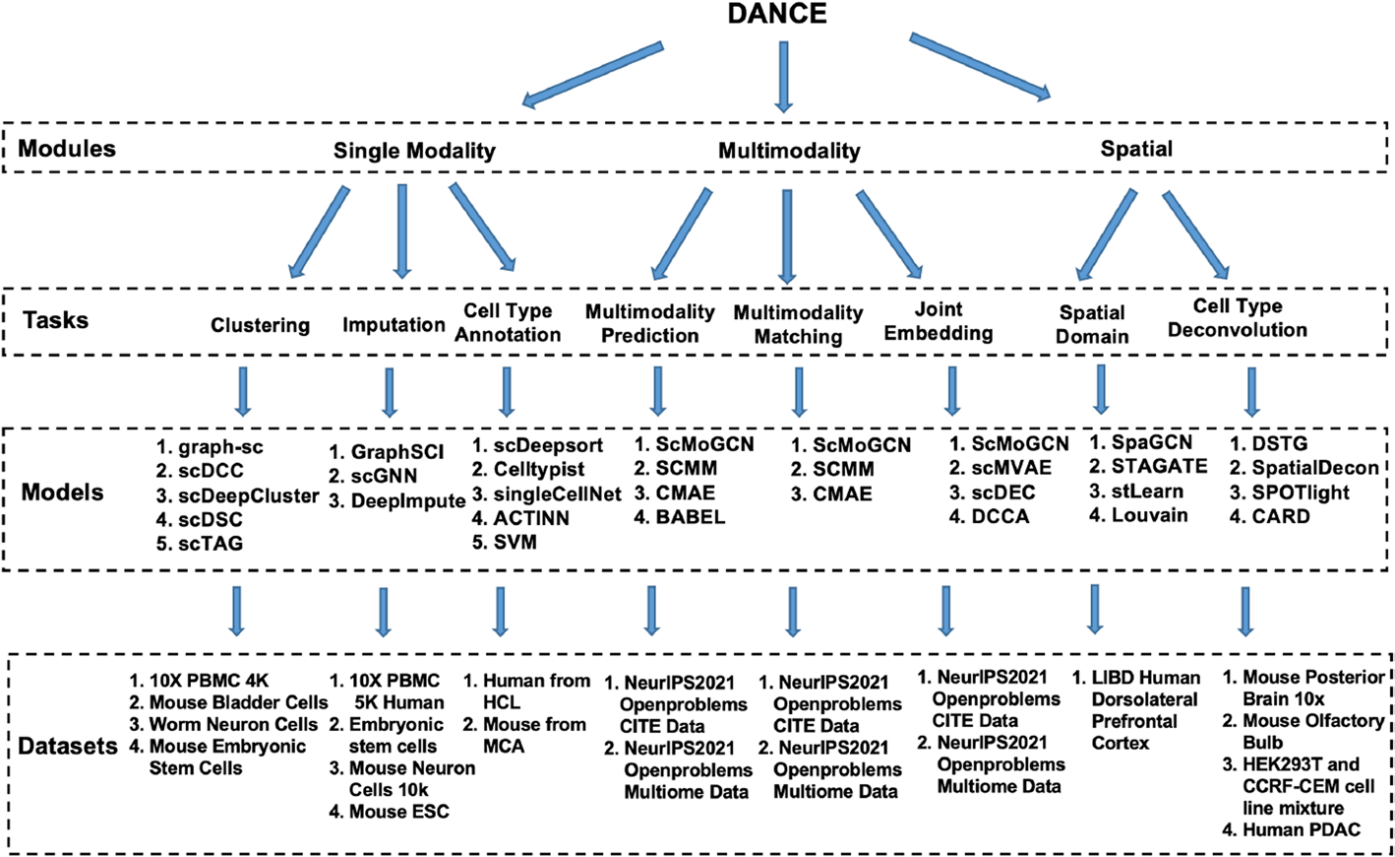
A summary of modules, tasks, models, and datasets supported by the DANCE package

As shown in Fig. 2, DANCE is capable of supporting modules of single modality, multimodality, and spatial transcriptomics. Under each module, we benchmark several tasks with popular models across standard datasets.

Here, we take the task of clustering in the module of single modality as an example. Various types of methods are implemented including GNN-based methods including graph-sc [25], scTAG [51], and scDSC [35] and AE-based methods including scDeepCluster [34] and scDCC [52]. To ensure a systematic evaluation and fair performance comparison of different models, several standard benchmark datasets such as 10X PBMC 4K [53], Mouse Bladder Cells [54], Worm Neuron Cells [55], and Mouse Embryonic Stem Cells [56] for the task are collected for evaluation. Currently, there are 3 modules, 8 tasks, 32 models, and 21 datasets supported by DANCE. Please refer to the “Methods” section for more details about supported models and datasets.

### Comparison with existing packages for single-cell analysis

DANCE is not only acting as a deep learning library to facilitate users’ model development but also as a benchmark platform for comprehensive evaluation. Table 1 summarizes the key differences between DANCE and existing single-cell libraries and toolkits. The highlights of DANCE are summarized as follows:

- *Comprehensive module coverage*: Squidpy [57] proposes an efficient and scalable infrastructure only for spatial omics analysis. DeepCell [58] forms a deep learning library for single-cell analysis but only biological images are covered. The library specializes in models for cell segmentation and cell tracking. Even though the popular Scanpy [59] provides a powerful tool for single-cell analysis spanning all modules, it focuses on the field of data preprocessing instead of modeling. Similarly, even though Seurat [10] touches on all three modules, its R language-based interface restricts its applicability for the development of deep learning methods due to limited R interface support within the deep learning community. Instead, DANCE supports all types of data preprocessing and modeling across all modules including single modality, multimodality, and spatial transcriptomics.
- *Deep learning infrastructure*: With the great increase in the number of single cells, classical methods [60, 61] cannot effectively enjoy the benefit from big single-cell data, while deep learning has been proven to be effective. Furthermore, deep learning techniques are also good at handling high dimensional data, which is common for single-cell data. Unfortunately, the backend framework of the well-known Seurat is R, which limits its potential in the deep learning community due to restricted R interface support in the deep learning community. Scanpy only contains classical methodologies for downstream tasks. Recently, scvi-tools [62] presents a Python library for deep probabilistic analysis of single-cell omics data. With 12 models, scvi-tools offers standardized access to 9 tasks. scvi-tools includes some deep learning methods but lacks the recent GNN-based methods. In terms of models, scvi-tools selects baselines with a concentration on statistical models according to their supporting data protocol. As a comparison, DANCE is a comprehensive deep learning library of single-cell analysis. Popular deep learning infrastructures like AEs [45] and GNNs are supported and applicable for all modules.
- *Standardized benchmarks*: To the best of our knowledge, DANCE is the first comprehensive benchmark platform covering all modules in single-cell analysis. A few unique features have been developed to achieve this goal. We first collect task-specific standard benchmark datasets and provide easy access to them by simply changing the parameter setting. Under each task, representative classical and deep learning algorithms are implemented as baselines. Those baselines are further fine-tuned on all collected benchmark datasets to reproduce similar or even better performance compared to original papers. To easily reproduce the results of our finetuned models, end users only need to run one command line where we wrap all super-parameters in advance to obtain reported performance.

**Table 1:** Comparison between DANCE and other popular single-cell libraries and toolkits

		Scanpy	Seurat	scvi-tools	DeepCell	Squidpy	DANCE
**Comprehensive module coverage**	Single modality	✓	✓	✓	✓	✗	✓
	Multimodality	✓	✓	✓	✗	✗	✓
	Spatial	✓	✓	✓	✗	✓	✓
**Deep learning infrastructure**	Classical deep learning	✗	✗	✓	✓	✓	✓
	GNNs	✗	✗	✗	✗	✓	✓
**Standardized benchmarks**	Benchmark datasets	✓	✓	✗	✓	✗	✓
	Task-specific algorithms	✓	✓	✗	✓	✗	✓
	Reproducible command lines	✗	✗	✗	✗	✗	✓

### Unified interface

**Fig. 3:**
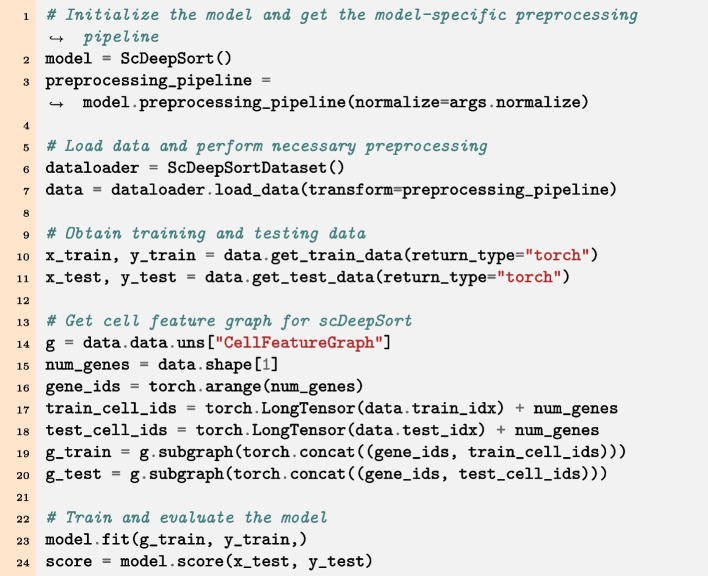
Consistent user experience

All models in DANCE are reimplemented in a unified development environment based on python language using Pytorch [29], DGL [30], and PyG [31] as backbone frameworks. What is more, all models in DANCE have generic interfaces for usage. As shown in Fig. 3, data loading is executed in a generic way via dataloader.load_data(), and model.preprocessing_pipeline() works for all datasets and models to specify model specific preprocessing functions. The interfaces of data.get_train_data() and data.get_test_data() are used to get training and test data respectively. For model training and evaluation, the unified interface for model training is model.fit(). Furthermore, model.score() acts as a generic interface to evaluate how well each model is. The metric of the score function depends on each task. Take scDeepSort [26] for an example, after fitting the model with chosen hyperparameters, we can access the performance of scDeepSort by calling the score function, which will return accuracy to indicate the quality of cell type annotation as a classification task.

### Performance showup

To build up a benchmark platform with the capability of systematic evaluations and fair comparisons of available methods, we first collect standard benchmark datasets by task. Then, we reimplement popular existing works for each task in a unified development environment based on the Python programming language with the Pytorch, DGL, and PyG frameworks as the backbone. Finally, we conduct exhaustive experiments of each reimplemented model on collected datasets. The data type supported in DANCE for benchmarking comes from single modality profiling (RNA, protein, and open chromatin) [1–9], multimodal profiling [10–14], to spatial transcriptomics [15–22]. Currently, DANCE supports three tasks in the single-modality module, three tasks in the multi-modality module, and two tasks in the spatial transcriptomics module.

#### Single-modality module―clustering

**Fig. 4:**
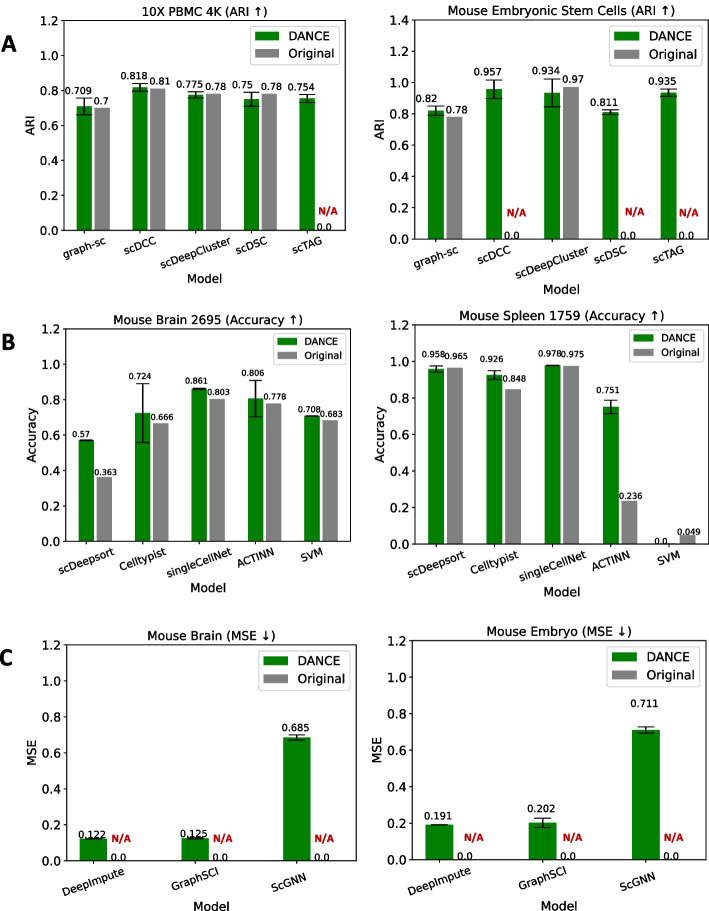
Performance comparison between our implementation and original implementation for supported tasks in the single-modality module. DANCE result represents the mean performance across 20 randomly chosen seeds, while the original result refers to the performance directly extracted from the original paper. **a** Clustering task. **b** Cell type annotation task. **c** Imputation task. Note: N/A indicates no performance report from the original paper

Clustering is a key component of single-cell analysis in the single-modality module. Researchers can distinguish between different cell types or cell type subgroups in the gene expression data using clustering. Adjusted Rand Index (ARI) is employed as an evaluation metric. Three GNN-based methods (graph-sc [25], scTAG [51], scDSC [35]) and two AE-based methods (scDeepCluster [34], scDCC [52]) have been reimplemented under this task. scDSC is deep structural clustering for single-cell RNA-seq data (scRNA-seq) using AEs and GNNs in conjunction. graph-sc and scTAG both convert scRNA-seq data to the cell-to-gene graph as an input for the graph encoder, while scTAG takes topology adaptive graph convolutional network (TAGCN) [63] as the graph encoder. scDeepCluster is a ZINB-based AE method for clustering. Similar model structure to scDeepCluster, scDCC additionally adds pairwise constraints into the loss function. Those five reimplemented models are evaluated on our collected four standard benchmarking datasets, which are 10X PBMC 4K [53], Mouse Bladder Cells [54], Worm Neuron Cells [55], and Mouse Embryonic Stem Cells [56]. There are 4271 cells and 16,653 genes with protocol as 10x Genomics in 10X PBMC 4K dataset, 2746 cells and 20,670 genes with protocol as Microwell-seq in Mouse Bladder Cells dataset, 4186 cells and 13,488 genes with protocol as sci-RNA-seq in Worm Neuron Cells dataset, and 2717 cells and 24,175 genes with protocol as Droplet Barcoding in Mouse Embryonic Stem Cells dataset. Figure 4a shows performance comparison between our implementation and the original implementation of five popular methods on 10X PBMC 4K and Mouse Embryonic Stem Cells datasets. We note that our graph-sc implementation increases slightly from 0.7 to 0.709 and from 0.78 to 0.82 on 10X PBMC 4K and Mouse Embryonic Stem Cells datasets respectively. scDCC performs similarly with the original implementation on the first dataset. On the other hand, we can also observe that our scDeepCluster achieves a similar performance to the original one on the first dataset but gets a worse performance on the second dataset since the variance among random seeds on the second dataset is large. What is more, scTAG in the original paper did not report its performance on both datasets. Instead, to have systematic evaluations, we fill the space of all missing reported performance. For the performance of five methods on more datasets, please refer to Additional file 5.

#### Single-modality module―cell type annotation

In the single-modality module, cell type annotation is to annotate the cell type of an individual cell by comparing the query data to annotated reference data (e.g., a single-cell atlas) or employing marker genes indicative of a particular cell type for annotation or modeling as supervised/semi-supervised learning task. Evaluation of model performance is based on prediction accuracy. Five existing works have been reimplemented under this task. scDeepsort [26] is a pre-trained cell type annotation method developed with a weighted GNN framework. Celltypist [64] is a multinomial logistic regression model for classification. SingleCellnet [65] is a random forest-based method, and support vector machine (SVM) [66] is a traditional support vector machine based method to enable the classification of scRNA-seq data. ACTINN [33] is a neural network-based method via multilayer perceptron. Two benchmark datasets have been collected for this task. HCL [67] dataset consists of 562,977 cells, while MCA [68] dataset consists of 201,764 cells. Figure 4b shows performance comparison between our implementation and the original implementation of such five popular methods on the MCA dataset (Mouse Brain 2695 and Mouse Spleen 1759). We can see that most of our implementation models outperform or match the original implementation on both Mouse Brain 2695 and Mouse Spleen 1759 datasets. scDeepsort outperforms the original implementation by a large margin on Mouse Brain 2695 while ACTINN outperforms the original implementation greatly on Mouse Spleen 1759. We also observe that the performance of our scDeepsort and ACTINN is lower than the reported performance from the paper on Mouse Kidney 203 in Additional file 5, which may be explained by the deviation from our implementation or the reported performance from the original paper. For performance comparison on more datasets, please refer to Additional file 5.

#### Single-modality module―imputation

In the single-modality module, imputation is to correct erroneous zeros by calculating plausible values for gene-cell pairs. For scRNA-seq data, imputation generates false count values for non-expressed genes, but for DNA methylation, imputation provides just the binary one or zero. Mean squared error (MSE) is used as an evaluation metric. Two GNN-based methods and one neural network-based method have been reimplemented under this task. scGNN [40] employs an integrative AE framework that combines gene regulatory signals for scRNA-seq gene expression imputation. GraphSCI [41] employs a graph autoencoder on a cell graph and reconstructs the input using the graph as additional input. DeepImpute [32] constructs multiple neural networks in parallel to infer target genes from an input collection of genes. Four benchmark datasets have been collected for benchmarking under the imputation task. 10X PBMC 5K [69] dataset consists of 5247 cells and 33,570 genes for each cell. Human Embryonic Stem Cells (Human ESC) [70] dataset consists of 758 cells and 17,826 genes for each cell. Mouse Neuron Cells 10k [69] dataset contains 11,843 cells and 31,053 genes for each cell. Mouse ESC [56] dataset is composed of 2717 cells and 24,175 genes for each cell. Figure 4c shows performance comparison on 10X PBMC 5K (Mouse Brain) and Mouse ESC (Mouse Embryo). It is obviously noticed that GNN-based methods like scGNN and GraphSCI outperform simple neural network methods like DeepImpute greatly on both datasets. The performance reports of all three models are missing on both standard benchmark datasets, and we also fill the gap for systematic evaluation.

#### Multimodality module―modality prediction

**Fig. 5:**
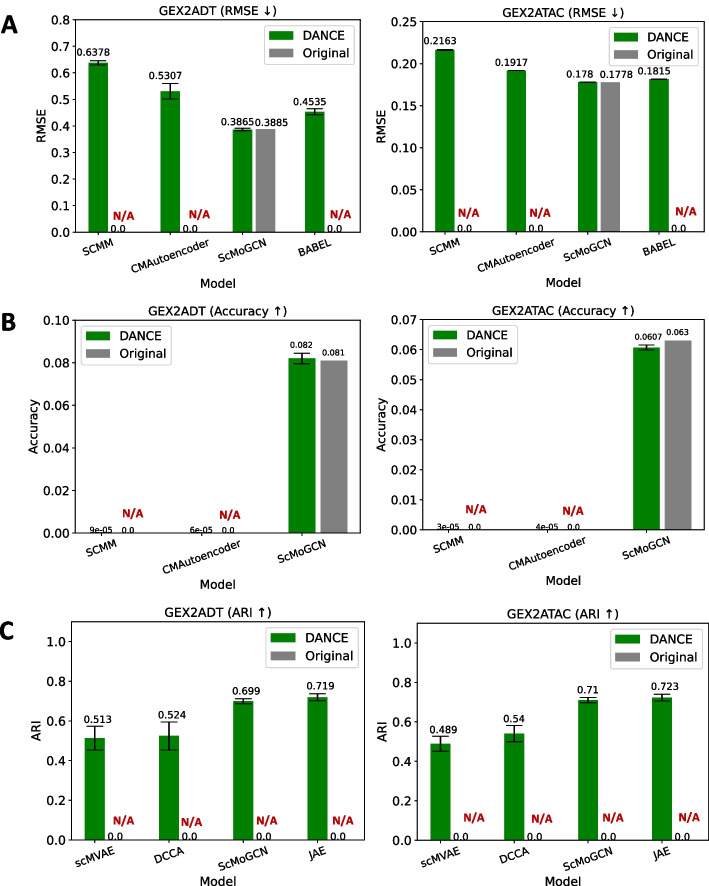
Performance comparison between our implementation and original implementation for supported tasks in the multi-modality module. DANCE result represents the mean performance across 20 randomly chosen seeds, while the original result refers to the performance directly extracted from the original paper. **a** Modality prediction task. **b** Modality matching task. **c** Joint embedding task. NOTE: N/A indicates no performance report from the original paper

In the multimodality module, modality prediction is to predict another modality like antibody-derived tags (ADT) given one modality like single-cell RNA-seq gene expression (GEX) for the same cell. Root mean square deviation (RMSE) is employed to evaluate how well the model performs on the task. Four deep learning-based methods have been reimplemented under this task. scMoGNN [11] for this task is an AE-based method to minimize the loss between one modality and another one. The input to the graph encoder is a cell-feature bipartite graph converted from the original input feature matrix. BABEL [36] trains two neural network-based encoders and two decoders to translate data from one modality to the other and reconstruct itself as well. Cross-modal autoencoders [37] uses AEs to map significantly distinct modalities (including pictures) to a common latent space. scMM [38] is a generative model which makes use of a mixture-of-experts (MoE) multimodal variational autoencoder (VAE) to investigate the latent dimensions associated with multimodal regulatory programs. Openproblems Neurips2021 competition datasets [71] have been collected for benchmarking under this task. Openproblems Neurips2021 CITE and Openproblems Neurips2021 Multiome contain 81,241 cells and 62,501 cells, respectively. As we can see from Fig. 5a, the GNN-based method scMoGNN outperforms other methods on both sub-task datasets GEX2ADT and GEX2ATAC. GEX2ADT means given GEX to predict ADT while GEX2ATAC means given GEX to predict ATAC. Most of the models have not been tested on those two datasets. We report all performances for a fair comparison. For more sub-task datasets like ADT2GEX and ATAC2GEX, please refer to Additional file 5.

#### Multimodality module―modality matching

In the multimodality module, modality matching is to match the profiles of each cell from different modalities. The task is evaluated by accuracy. Same with modality prediction, scMoGNN, scMM, and cross-modal autoencoders have been reimplemented with modifications to be suitable for this task. Details of modifications about three models can be found in Additional file 1. The same benchmark datasets with modality prediction have been benchmarked for this task. Similar finding on modality prediction, we can see that GNN-based method scMoGNN outperforms other methods by a huge margin on both sub-task datasets in Fig. 5b.

#### Multimodality module―joint embedding

In the multimodality module, joint embedding is to learn joint embedding from multiple modalities like GEX and ADT. ARI is employed as an evaluation metric. scMoGNN has been adjusted and implemented to solve this problem. In addition, JAE is a typical AE architecture with an encoder and a decoder, an adapted model from scDEC [72]. scMVAE [39] simultaneously employs three learning methodologies to discover the distribution of multi-omics: product of experts (PoE), neural networks, and concatenation of multi-omics features. DCCA [39] is a VAE-based method. Each VAE undergoes independent training with each modality. Two VAEs are then trained in tandem to optimize the similarity between two latent spaces. Same benchmark datasets with the previous two tasks, as shown in Fig. 5c, GNN-based method scMoGNN resides in TOP 2 on both sub-task datasets. All methods have not been evaluated on both datasets, and we fill the gap for systematic evaluation.

To make the evaluation more systematic and comprehensive, we additional support biology conservation metrics and batch removal metrics under this task in addition to ARI metric. For biology conservation evaluation, normalized mutual information (NMI) metric is adopted to compare the overlap of two clusterings, and cell cycle conservation (Cc_cons) score metric is served as a proxy for the preservation of the signal associated with gene programs during data integration. For batch removal evaluation, ASW batch (ASW_batch) metric is used to quantify batch mixing by taking into account the incompatibility of batch labels per cell type cluster, and graph connectivity (Graph_conn) metric is employed to determine whether cells of the same kind from various batches are embedded close to one another. The benchmarking results for those metrics can be found in Additional file 5.

#### Spatial transcriptomics module―spatial domain identification

**Fig. 6:**
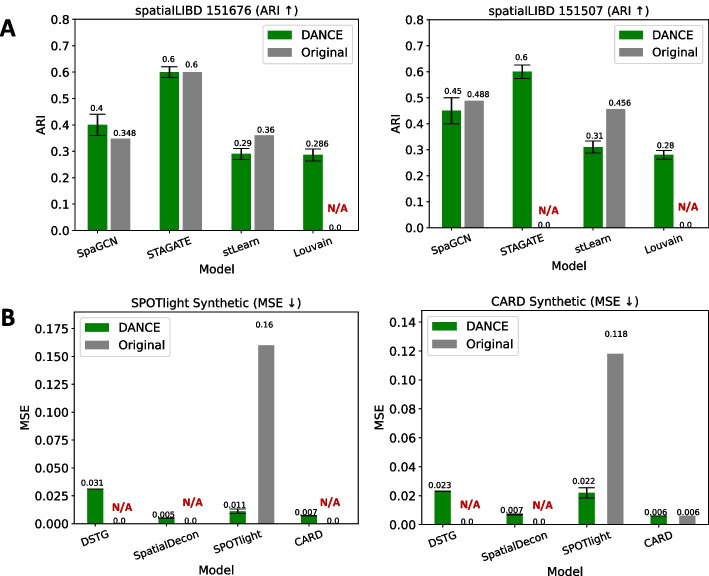
Performance comparison between our implementation and original implementation for supported tasks in the spatial transcriptomics module. DANCE result represents the mean performance across 20 randomly chosen seeds, while the original result refers to the performance directly extracted from the original paper. **a** Spatial domain identification task. **b** Cell type deconvolution task. Note: N/A indicates no performance report from the original paper

In the spatial transcriptomics module, spatial domain identification seeks to cluster spatial data into a series of meaningful groups. Each group discovered is regarded as a spatial domain. ARI is employed as an evaluation metric for this task. Two GNN-based methods and two traditional methods have been reimplemented. SpaGCN [42] is a GCN-based method for locating geographic domains and variable genes by integrating gene expression and histology. STAGATE [43] is a graph attention method based on AE framework that learns low-dimensional latent embeddings using gene expression and geographical data. Louvain [73] is an iterative strategy for optimizing modularity that is used for network community detection. stLearn [74] does unsupervised clustering on the data that has been normalized by SMEs in order to aggregate similar regions into clusters and find sub-clustering based on the geographic separation of clusters within the tissue. The most popular benchmark dataset LIBD human dorsolateral prefrontal cortex [75] has been collected, and it contains 12 slices. As shown in Fig. 6a, it is obvious that two GNN-based methods SpaGCN and STAGATE outperform others on both slices. Our STAGATE achieves similar results to the original implementation. For average performance on all 12 slices, please refer to Additional file 5.

#### Spatial transcriptomics module―cell type deconvolution

In the spatial transcriptomics module, cell type deconvolution is to estimate cell type proportions in spatial transcriptomic data. MSE is used as an evaluation metric. One GNN-based method and four traditional methods have been reimplemented under this task. DSTG [44] deconvolutes spatial transcriptomic data using graph-based convolutional networks in order to precisely deconvolve the observed gene expressions at each location and restore their cell constitutions. SPOTlight [76] is a computational method that integrates ST and scRNA-seq data to infer the location of cell types and states within a complicated tissue. It is centered on a seeded non-negative matrix factorization (NMF) regression, which is initialized with cell type marker genes and non-negative least squares (NNLS) to deconvolute ST capture locations (spots). SpatialDecon [77] harnesses log-normal regression and modeling background to quantify cell populations defined by single-cell sequencing within the regions of spatial gene expression. CARD [78] is a conditional autoregressive-based deconvolution that combines cell type-specific expression information from scRNA-seq with correlation in cell type composition across tissue locations. Four standard benchmark datasets have been collected for this task. Mouse Posterior Brain [79] contains 3353 spots, Mouse Olfactory Bulb [80] consists of 1185 spots, HEK293T and CCRF-CEM [81] contain 56 mixtures, and Human PDAC [82] includes 3353 spots. As shown in Fig. 6b, with our reimplementation, SPOTlight improves from 0.16 to 0.011 on Mouse Posterior Brain (SPOTlight Synthetic) and from 0.118 to 0.022 on Mouse Olfactory Bulb (CARD Synthetic). For performance comparison on more datasets, please refer to Additional file 5.

### GPU acceleration

**Fig. 7:**
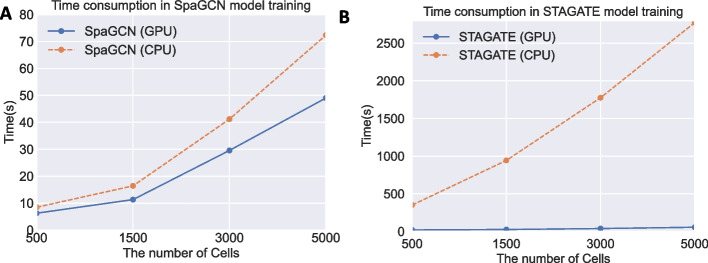
The comparison of time consumption in model training between implementations with CPU and GPU support. The original implementation comes with CPU but not GPU acceleration while ours is enhanced with GPU support. The SpaGCN and STAGATE with GPU is the implementation in our DANCE. **a** SpaGCN model in spatial domain identification task. **b** STAGATE model in spatial domain identification task

In addition to the mentioned similar or better performance of our implementation, we also demonstrate the computational improvement using the GPU implementations in DANCE that are not supported by the original sources. We take SpaGCN and STAGATE models in the task of spatial domain identification as examples. All experiments in this demonstration are carried out on the device with the same memory of 16GB. For GPU supported environment, we use a single node with amd20-v100 while we use a single node with amd20 for CPU running testing. The reported time consumption is from the average of five runs. Following the original papers, SpaGCN is trained with 500 epochs while STAGATE is trained with 2000 epochs. As shown in Fig. 7a, as the number of training cells increases, the computational advantage of our SpaGCN with GPU support becomes more appreciable from the perspective of training time. This phenomenon is more obvious in the STAGATE model as shown in Fig. 7b. The training time consumption for our STAGATE is 21 s, 26 s, 39 s, and 56 s corresponding to the training cells of 500, 1500, 3000, and 5000 cells, which are 16x, 36x, 45x, and 50x times speedup of the STAGATE implementation with CPU support, respectively. As the number of training cells continues to increase, our acceleration factor will expand greatly.

### Easy reproduction

Due to the lack of a publicly available codebase and variances in programming languages, the diversity and complexity of deep learning methods make it difficult for researchers to reproduce the results from the original papers. Another reason specifically for deep learning approaches that cannot be overlooked is hyperparameter tuning. Hyperparameter tuning is to find a set of optimal hyperparameter values for a learning algorithm while applying this optimized algorithm to any data set. This combination of hyperparameters maximizes the performance of the model on a specific dataset. The hyperparameters here are not only model-specific parameters but also the common neural network parameters that must be tuned, such as the number of neurons in the neural network layer, activation function selection, weight decay, and learning rate.

Note that hyperparameter tuning is an empirical task. Based on our many years of tuning experience for deep learning approaches, we execute exhaustive hyperparameter tunning experiments to get optimal model performance on a certain dataset offline and then wrap those optimal hyperparameters values into one command line for reproduction. In such case, users only need to run one command line to obtain reported performance. We have recorded all command lines at the end of model usage example files in DANCE package (https://github.com/OmicsML/dance). The below introduces a few examples of DSTG [44] model on certain benchmark datasets in the task of cell type deconvolution.

DSTG model on CARD synthetic benchmark dataset:
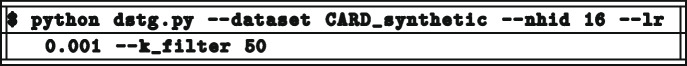


DSTG model on GSE174746 benchmark dataset: 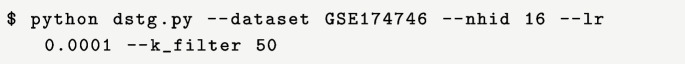


DSTG model on SPOTLight synthetic benchmark dataset: 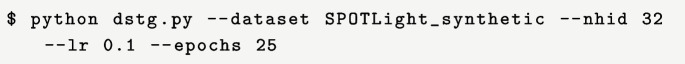


In addition, if a newly model is added into DANCE, end users can easily execute the command line by specifying searching space of any interested parameter in the command line for hyperparameter tunning.

### Extensible benchmarking

The DANCE platform is the standard, flexible, and extensible benchmark platform for accessing and assessing computational methods across a spectrum of benchmark datasets for a variety of single-cell analysis tasks. Note that even though the limited tasks, models, and benchmark datasets are supported in DANCE, DANCE is being implemented in a highly modular manner, allowing it to be readily expanded and maintained by a community.

We will also keep contributing to include more tasks, models, and benchmark datasets. Our goal is to build up a deep learning and benchmarking community. A HANDS-ON LAB, LIVE TUTORIAL was hosted by the DANCE team in June this year via Zoom to guide users on how to use DANCE including DANCE environment setup, data loading and processing, basic deep learning framework walk-through, example methods providing a detailed, step-by-step tutorial for each task. We have provided a detailed tutorial notebook that can be launched from Google Colab in a dedicated repository (https://github.com/OmicsML/dance-tutorials/blob/tutorial-v1/dance_tutorial.ipynb). We encourage the community to contribute to this extensible benchmark platform to facilitate the overall advancement of single-cell analysis research. For open-source contributions, please refer to Additional file 4 for more details about contribution instructions in DANCE.

## Conclusions

In the realm of single-cell analysis, computational approaches have brought an increasing number of fantastic prospects for innovation and invention. Meanwhile, it also presents enormous hurdles to reproducing the results of these models due to their diversity and complexity. In addition, the lack of gold-standard benchmark datasets, metrics, and implementations prevents systematic evaluations and fair comparisons of available methods. Thus, we introduce the DANCE platform, the first standard, generic, and extensible benchmark platform for accessing and evaluating computational methods across the spectrum of benchmark datasets for numerous single-cell analysis tasks. Currently, DANCE supports 3 modules and 8 popular tasks with 32 state-of-art methods on 21 benchmark datasets. The performance of our reimplemented models is equivalent or even superior to that of the models in the original papers. We find that the majority of existing works have only reported their performance on limited datasets and in comparison with insufficient methods. We also find that the majority of the existing works cannot consistently perform well through all our collected benchmark datasets. Those prove that comprehensive benchmark datasets and metric evaluation are highly desired in this community. Moreover, we implement all models in a unified development environment based on the python language with Pytorch, DGL, and PyG as backbone frameworks. The interfaces across tasks to download data, read training/test data and train/test models are all unified. Both will greatly facilitate further DANCE development and easy maintenance, and provide a consistent user experience.

Another highlight of DANCE is the easy reproducibility of models. For each task, each implemented method in DANCE is tuned on all gathered standard benchmarks using a grid search to obtain the optimal model, and the corresponding hyperparameters are recorded in a single command line for easy user reproducibility. Last but not the least, due to the nature of extensible feature of our DANCE platform, more additional single-cell analysis tasks, models, and benchmark datasets are easily added and supported into DANCE platform to further enhance the significance and practical utility of the DANCE.

## Methods

### DANCE implementation and design overview

#### Environment requirements and setup

DANCE works on python $\ge 3.8$ and Pytorch $\ge 1.11.0$. All dependencies are listed in Additional file 2. After cloning this repository, run setup.py to install DANCE into the local python environment or install it directly from pip install as below:
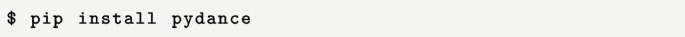


#### The architecture design and implementation

**Fig. 8:**
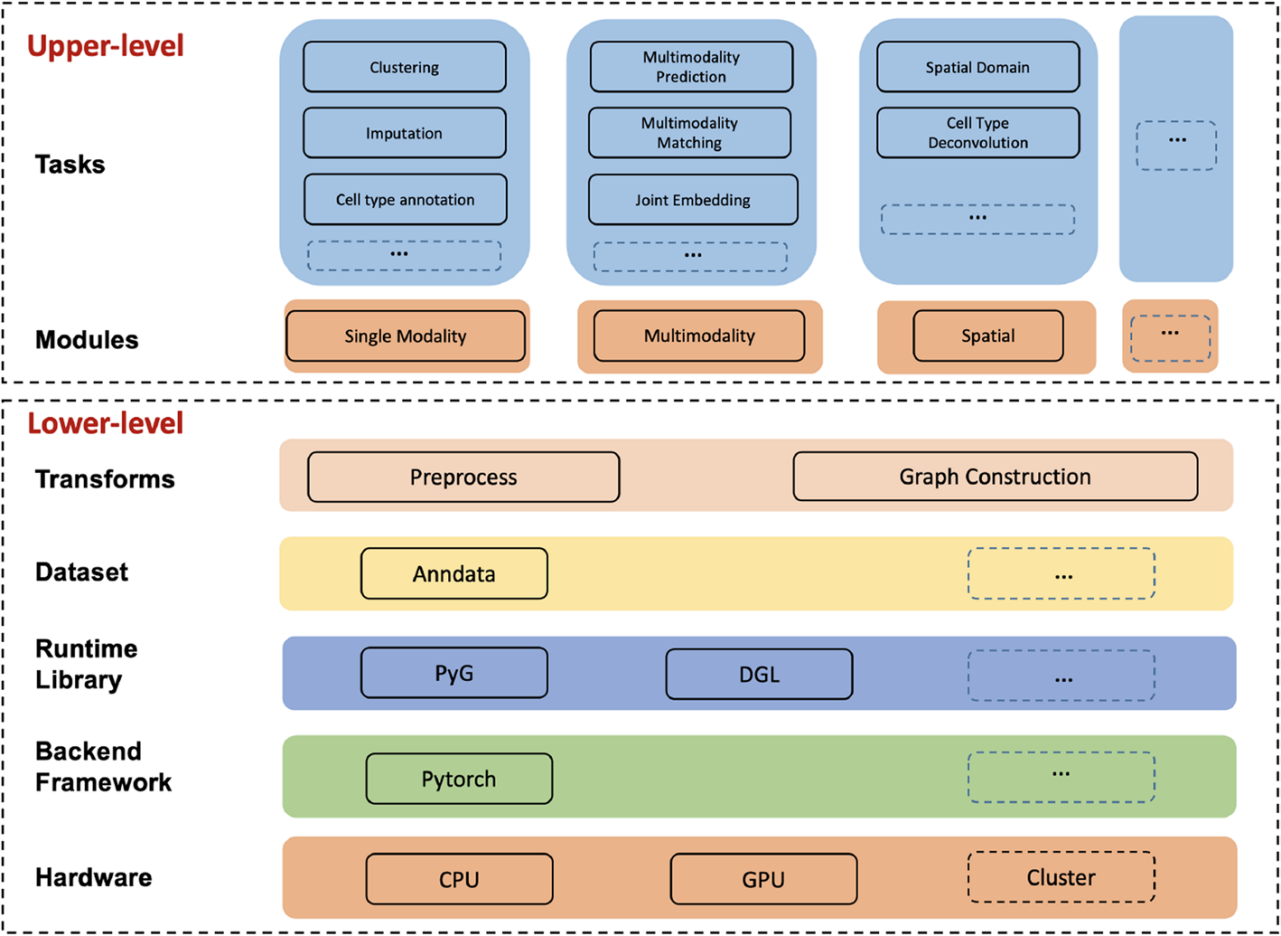
The architecture of DANCE package

Figure 8 provides an overall design of the architecture of the DANCE package. The DANCE package consists of two key components: lower-level infrastructure and upper-level task development.

***Lower-level infrastructure***

From the hardware perspective, CPU running is supported for all methods developed in DANCE. In addition, for deep learning-based methods, we also support GPU running to accelerate the training process, especially for large-scale datasets. In the future, cluster running for deep learning methods would be also developed to support model training across multiple GPUs. The backbone framework in DANCE is Pytorch [29], which is used for high-performance deep learning model development. To support various methods for deep learning on graphs and other irregular structures, we take both DGL [30] and PyG [31] as graph engines in DANCE. Various types of preprocessing functionalities are provided in the Transforms folder to process data before model training. For methods based on GNNs, we also support distinct ways of graph construction to convert cell-gene data like RNA sequencing (RNA-seq) to cell-cell, cell-gene, and gene-gene graphs. What is more, spatial coordinates and image features of single cells can be also extracted to help construct graphs for spatial transcriptomics. Those lower-level interfaces are helpful for developers to build their models on downstream tasks without building “wheels” from scratch.

***Upper-level task development***

Based on the infrastructure described above, individual modules and tasks can be further defined and developed. Currently, we support tasks under single modality profiling, multimodal profiling, and spatial transcriptomics modules, which correspond to three stages of single-cell technology development. Under each module, classic tasks are covered, and representative methods are implemented through the evaluation of several standard benchmarks. Note that upper-level task development is highly flexible and extensible. This indicates that users can readily extend their new modules, tasks, models, and datasets into the existing repository of DANCE.

### Benchmark datasets supported in DANCE

All supported benchmark datasets across 8 tasks in DANCE are summarized in Table 2. For each supported dataset, we list what type of species and tissue it is about, dataset dimensions including the number of cells and genes, and also the protocol about how to generate the dataset for reference. In the column of “Availability,” the dataset link is provided once you click the reference.

**Table 2:** A summary of all supported benchmark datasets in DANCE

Module	Task	Dataset	Species and tissue	Dataset dimensions	Protocol	Availability
**Single modality**	**Imputation**	10X PBMC 5K	Human, PBMC	5247 cells	10x Genomics	[69]
				33,570 genes		
		Human Embryonic Stem Cells (ESC)	Human, ESC	758 cells	Illumina HiSeq 2500	[70]
				17,826 genes		
		Mouse Neuron Cells 10k	Mouse, Neuron	11,843 cells	10x Genomics	[69]
				31,053 genes		
		Mouse ESC	Mouse, Neuron	2717 cells	Droplet Barcoding	[56]
				24,175 genes		
	**Cell type annotation**	HCL	Human	562,977 cells	Smart-seq2	[67]
				56 tissues		
		MCA	Mouse	201,764 cells	Smart-seq2	[68]
				32 tissues		
	**Clustering**	10X PBMC 4K	Human, PBMC	4271 cells	10x Genomics	[53]
				16,653 genes		
		Mouse Bladder Cells	Mouse, Bladder	2746 cells	Microwell-seq	[54]
				20,670 genes		
		Worm Neuron Cells	Worm, Nerve	4186 cells	sci-RNA-seq	[55]
				13,488 genes		
		Mouse Embryonic Stem Cells	Mouse, Embryo	2717 cells	Droplet Barcoding	[56]
				24,175 genes		
**Multimodality**	**Modality prediction**	Openproblems Neurips2021 CITE	Human, BMMC	81,241 cells	10X TotalSeq B	[71]
				13,953 genes		
				134 surface proteins		
		Openproblems Neurips2021 Multiome	Human, BMMC	62,501 cells	10X Multiome	[71]
				13,431 genes		
				116,490 peaks		
	**Modality matching**	Openproblems Neurips2021 CITE	Human, BMMC	81,241 cells	10X TotalSeq B	[71]
				13,953 genes		
				134 surface proteins		
		Openproblems Neurips2021 Multiome	Human, BMMC	62,501 cells	10X Multiome	[71]
				13,431 genes		
				116,490 peaks		
	**Joint embedding**	Openproblems Neurips2021 CITE	Human, BMMC	81,241 cells	10X TotalSeq B	[71]
				13,953 genes		
				134 surface proteins		
		Openproblems Neurips2021 Multiome	Human, BMMC	62,501 cells	10X Multiome	[71]
				13,431 genes		
				116,490 peaks		
**Spatial**	**Spatial domain**	LIBD Human Dorsolateral Prefrontal Cortex	Human, Dorsolateral prefrontal cortex	12 slices	10X Visium	[75]
				Slice 151673:		
				3639 spots		
				33,538 genes		
	**Cell type deconvolution**	Mouse Posterior Brain	Mouse, Posterior brain	3353 spots	10X Visium	[79]
				31,053 genes		
		Mouse Olfactory Bulb	Mouse, Olfactory bulb	1185 spots	10X Visium	[80]
				11,176 genes		
		HEK293T and CCRF-CEM	Human	56 mixtures	NanoString GeoMx	[81]
				1414 genes		
		Human PDAC	Human, Pancreas	3353 spots	Spatial Transcriptomics	[82]
				31,053 genes		

### Task definition and evaluation metrics

#### Single-modality module―imputation

The goal of imputation for scRNA-seq data is to address artificial zeros in scRNA-seq data generated during the sequencing process systematically or by chance due to technological limitations. Imputation aims at correcting these artificial zeros by filling in realistic values that reflect true biological gene expressions [83]. Thus, a good imputation method should be able to distinguish artificial zeros from biologically true zeros and recover true expressions for artificial zeros. As the corresponding biologically true expression values are unavailable for entries of artificial zeros in the gene-cell matrix, dropouts are simulated for benchmarking such that metrics such as cosine similarity, correlations, or MSE-related metrics can then be used to evaluate imputation algorithms.

#### Single-modality module―cell type annotation

Cell type annotation targets applying statistics of cellular properties to infer cell types. Given the gene expression of several cell types, for each cell with a certain single-cell expression matrix, the degree of similarity can be calculated. Based on the optimal similarity result, the cell type can then be inferred. In DANCE, we support 5 models that establish measurements of evaluating the similarity of gene expression profiles of unknown cells to gene expression matrices of known cell types. The model performance is evaluated by prediction accuracy.

#### Single-modality module―clustering

Clustering is a crucial part of single-cell analysis. With clustering, researchers can identify cell types or cell type subgroups within the gene expression data. In the clustering task, we now support 5 models. The first 3 models are GNN based, and the later 2 models are non-GNN based with AE as the backbone. The clustering performance is evaluated by ARI.

#### Multimodality module―modality prediction

Modality prediction is to predict features of a target modality from features of an input modality. The evaluation is based on RMSE between ground-truth features and prediction. In this task, DANCE supports 4 models. All of them are deep learning models, one of which is based on graph neural networks.

#### Multimodality module―modality matching

The objective of the modality matching task is to identify cell correspondence across modalities. To be concrete, we separate each modality of the jointly profiled dataset into a subset, and the order of cells in each subset is disturbed. In the training dataset, the cell correspondence labels between subsets are given. While in the testing data, the correspondence is not given. The model needs to learn to identify cell correspondence from the labeled training data and evaluate it on the testing data. To provide a more flexible protocol, the model output is adapted to a matching score matrix $\textbf{S} \in \mathbb {R}^{n \times n}$, where *n* is the number of cells, and $\textbf{S}_{i,j}$ is the probability that cell *i* from one modality corresponds to cell *j* from the other modality. Therefore, $\textbf{S}$ is a non-negative matrix where each row sums to 1. As metrics, we compute the average probability assigned to the correct matching. In this module, DANCE now supports 3 models. All of them are deep learning models, one of which is based on GNNs.

#### Multimodality module―joint embedding

Joint embedding aims to encode features from two modalities into a low-dimensional joint latent space. To be consistent with the NeurIPS competition [84], we set the latent dimension size less than or equal to 100. For the evaluation, currently, we only support normalized mutual information(NMI) and ARI with the k-means clustering as metrics in our DANCE package. These metrics evaluate the consistency between latent clusters and the ground-truth cell type labels. More comprehensive metrics were introduced in the competition, and we are going to incorporate them into our package in the future. In this task, DANCE now supports 4 models. All of them are deep learning models, one of which is a GNN.

#### Spatial transcriptomics module―spatial domain

In spatial transcriptomics, the spatial data is referring to spots with *x*,*y* coordinates, and each spot captures several cells. The objective of the spatial domain is to partition the spatial data into meaningful clusters. Each cluster uncovered by this analysis is regarded as a spatial domain. Spots in the same spatial area are comparable and consistent in gene expression and histology, but spots in different spatial regions are distinct [85]. For evaluation, ARI [86] is utilized to compare the efficacy of various clustering techniques. It computes the similarity between the algorithm-predicted clustering labels and the actual labels. In the spatial domain task, DANCE supports 4 models including 2 GNN-based models and 2 traditional models.

#### Spatial transcriptomics module―cell type deconvolution

Cell type deconvolution is the task of estimating cell type composition in cell pools from their aggregate transcriptomic information. This is a type of inverse problem, as we are trying to determine the signal of individual cell types from aggregated readings across multiple cell types. Moreover, due to the nature of the spatial (or bulk) transcriptomics profiling technologies, the true cell type compositions are most often not given. For the task of cell type deconvolution, DANCE supports 4 models, one GNN-based model, and 3 non-GNN-based models with classical regression models as their backbone. The performance is evaluated by MSE.

### Reimplemented models in DANCE

DANCE currently supports total 32 models, which are 3 models in imputation, 5 models in cell type annotation, 5 models in clustering, 4 models in modality prediction, 3 models in modality matching, 4 models in joint embedding, 4 models in the spatial domain, and 4 models in cell type deconvolution. The below will briefly introduce each method. For more details about each model, please refer to Additional file 1.

#### Single-modality module―imputation

dance.modules.single_modality.imputation.deepimpute

DeepImpute [32] builds multiple neural networks in parallel to impute target genes using a set of input genes.

dance.modules.single_modality.imputation.scgnn

scGNN [40] uses an integrative autoencoder framework for scRNA-seq gene expression imputation that incorporates gene regulatory signals (TRS).

dance.modules.single_modality.imputation.graphsci

GraphSCI [41] is a GNN-based method to impute scRNA-seq data expressions. It uses two autoencoders: one being a graph autoencoder on a cell graph, and the other reconstructs the input using the graph as additional input.

#### Single-modality module―cell type annotation

dance.modules.single_modality.cell_type_annotation.scdeepsort

 Scdeepsort [26] a pre-trained cell type annotation method. It is developed with a weighted GNN framework and then trained on two embedded high-quality scRNA-seq atlases containing 764,741 cells from 88 human and animal tissues.

dance.modules.single_modality.cell_type_annotation.celltypist

 Celltypist [64] is a multinomial logistic regression classifier with stochastic gradient descent learning.

dance.modules.single_modality.cell_type_annotation.singlecellnet

 SingleCellnet [65] revamped the random forest classifier method to enable the classification of scRNA-seq data cross platforms and cross-species. It sends the input features into a number of decision tree classifiers and uses majority voting to make predictions.

dance.modules.single_modality.cell_type_annotation.actinn

 ACTINN [33] proposes a neural network-based model for cell type annotation. It applies multilayer perceptron for the identification of cell types.

dance.modules.single_modality.cell_type_annotation.svm

 SVM is widely adopted as a benchmark in many studies [26, 66]. It works by mapping data to a high-dimensional feature space so that data points can be categorized even when the data are not linearly distinct.

#### Single-modality module―clustering

dance.modules.single_modality.clustering.scdeepcluster

 scDeepCluster [34] is a ZINB-based AE method for clustering.

dance.modules.single_modality.clustering.scdcc

 scDCC [52] shares the same model structure as scDeepCluster. In the training process, pairwise constraints are integrated into the loss function.

dance.modules.single_modality.clustering.graphsc

 graph-sc [25] is GNN-based method for clustering scRNA-seq data by constructing gene-to-cell graph as the input of graph autoencoder.

dance.modules.single_modality.clustering.sctag

 scTAG [51] first generates a K-nearest neighbor cell-to-cell graph. It then adopts a ZINB-based graph autoencoder to process it, which takes TAGCN [63] as the graph encoder.

dance.modules.single_modality.clustering.scdsc

 scDSC [35] is deep structural clustering for single-cell RNA-seq data jointly through autoencoder and graph neural network.

#### Multimodality module―modality prediction

dance.modules.multi_modality.predict_modality.scmogcn

 scMoGNN [11] is a GNN-based method where the input feature matrix is converted into a cell-feature bipartite graph, where each node represents a cell or feature.

dance.modules.multi_modality.predict_modality.babel

 BABEL [36] trains two neural-network-based encoders and two decoders on the paired data to translate data from one modality to the other and to reconstruct itself, thus eventually obtaining shared embedding.

dance.modules.multi_modality.predict_modality.cmae

 Cross-modal autoencoders [37] use AEs to map vastly different modalities (including images) to a shared latent space.

dance.modules.multi_modality.predict_modality.scmm

 scMM [38] leverages a MoE multimodal VAE [87] to explore the latent dimensions that associate with multimodal regulatory programs.

#### Multimodality module―modality matching

dance.modules.multi_modality.match_modality.scmogcn

 The overall structure of scMoGNN in the modality matching task is the same as in the modality prediction task. However, in the modality prediction task, the input is only one modality, while in the modality matching task, features of two modalities are given altogether. Therefore, scMoGMM constructs two graphs for two modalities respectively.

dance.modules.multi_modality.match_modality.cmae

 The overall structure of cross-modal autoencoders is the same as in the modality prediction task, where we implement encoders and decoders for all the modalities. Hereby, in the modality matching task, we directly utilize the latent space instead of using a decoder to generate target modality.

dance.modules.multi_modality.match_modality.scmm

 The overall structure of scMM is the same as in the modality prediction task, where we implemented a neural network encoder for each modality to estimate the variational posterior. In the modality matching task, we hereby take the latent vectors generated by encoders as the source for matching.

#### Multimodality module―joint embedding

dance.modules.multi_modality.joint_embedding.scmogcn

 The overall structure of scMoGNN in the joint embedding task is still similar to what is shown in the modality prediction task. However, different from previous tasks, here scMoGNN first reduces the input dimension. Next, the preprocessed features of two modalities are concatenated and jointly considered as feature nodes in the graph construction. scMoGNN is further trained by minimizing a reconstruction loss, a cell type auxiliary loss, and a regularization loss.

dance.modules.multi_modality.joint_embedding.jae

 JAE is an adapted model from scDEC [72]. It is proposed by the authors of scDEC in the NeurIPS competition [84] to better leverage cell annotations. Formally, JAE follows the typical AE architecture with an encoder and a decoder.

dance.modules.multi_modality.joint_embedding.scmvae

 scMVAE [39] learns the distribution of multi-omics via three learning strategies simultaneously: PoE, neural networks, and concatenation of multi-omics features.

dance.modules.multi_modality.joint_embedding.dcca

 In DCCA [39], each modality is modeled by a VAE. Each VAE is first trained separately with each modality. Then, two VAEs are trained together to maximize the similarity between two latent spaces.

#### Spatial transcriptomics module―spatial domain

dance.modules.spatial.spatial_domain.spagcn

 SpaGCN [42] is a GCN-based method via integrating gene expression and histology to find spatial domains and variable genes.

dance.modules.spatial.spatial_domain.stagate

 STAGATE [43] is a graph attention-based autoencoder [45] to learn low-dimensional latent embeddings from gene expression and spatial information.

dance.modules.spatial.spatial_domain.louvain

 Louvain [73] is an iterative modularity optimization method for network community detection.

dance.modules.spatial.spatial_domain.stlearn

 stLearn [74] performs unsupervised clustering on SME-normalized data to group similar areas into clusters and discover sub-clustering alternatives based on the geographic separation of clusters inside the tissue.

#### Spatial transcriptomics module―cell type deconvolution

dance.modules.spatial.cell_type_deconvo.dstg

 DSTG [44] is a GCN-based method whose graph is constructed on mutual nearest neighbors of low-dimensional embeddings of simulated and real mixed-cell data.

dance.modules.spatial.cell_type_deconvo.spotlight

 SPOTlight [76] is an extension of NMFReg, with non-negative matrix factorization applied to both the scRNA reference matrix and the mixed-cell expression matrix.

dance.modules.spatial.cell_type_deconvo.spatialdecon

SpatialDecon [77] is a non-negative linear regression-based method that assumes a log-normal multiplicative error model between the mixed-cell data and a cell-profile (signature) matrix.

dance.modules.spatial.cell_type_deconvo.card

CARD [78] applies a conditional autoregressive (CAR) assumption on the coefficients of the classical non-negative linear model between the mixed-cell expression and a cell-profile matrix, constructed from reference scRNA-seq.

### Supplementary Information

**Additional file 1.** Appendix A — Details About Supported Models in DANCE.

**Additional file 2.** Appendix B — Environment Dependencies in DANCE.

**Additional file 3.** Appendix C — Codebase Structure Tree in DANCE.

**Additional file 4.** Appendix D — Contribution Instructions in DANCE.

**Additional file 5.** Appendix E — More Performance Showup in DANCE.

**Additional file 6.** Review history.

## Data Availability

DANCE’s open-source code is maintained on GitHub (https://github.com/OmicsML/dance) [88] and published under the BSD2 license. It is also deposited to Zenodo (https://zenodo.org/records/10648047) with assigned DOI: 10.5281/zenodo.10648046 [89]. The online documentation is available at (https://pydance.readthedocs.io/en/latest/) [90]. DANCE is released via the Python packaging index: https://pypi.org/project/pydance/. All collected benchmark datasets used in this study are available from Science Data Bank (https://www.scidb.cn/en/s/nmA7fy#p4) with assigned DOI: 10.1101/2022.10.19.512741 [91].
