# Supplementary material for: DANCE: a deep learning library and benchmark platform for single-cell analysis
Source: Genome Biol. 2024 Mar 19;25:72. doi: 10.1186/s13059-024-03211-z (PMC10949782; doi:10.1186/s13059-024-03211-z)
Supplement: Supplementary file 1 — Additional file 1. Appendix A — Details About Supported Models in DANCE. [file 13059_2024_3211_MOESM1_ESM.pdf]

Appendix A: Details About Supported Models in DANCE

A.1 Single Modality Module

A.1.1 Imputation

dance.modules.single_modality.imputation.deepimpute DeepImpute [32] builds multiple neural networks in parallel to impute target genes using a set of input genes. Given a scRNA-seq matrix X, target genes, i.e, genes to be imputed, are selected based on the variance over mean ratio, which are split into N random subsets. Each subset corresponds to a neural network consisting of two layers: a dense layer and a dropout layer with ReLu and softplus as activations, respectively. The inputs to each neural network are genes highly correlated with corresponding target genes based on Pearson's correlation coefficient. The loss function is the Weighted mean squared error (MSE).

dance.modules.single_modality.imputation.scgnn scGNN [40] uses an integrative autoencoder framework for scRNA-seq gene expression imputation that incorporates gene regulatory signals (TRS). It includes a feature autoencoder, a graph autoencoder, and a cluster autoencoder that are trained iteratively, whose outputs are used in the final imputation autoencoder to recover gene expressions.
scgnn uses left-truncated mixed Gaussian (LTMG) to account for regulatory signals. The normalized expression for a gene is modeled by a mixture of k Gaussian distributions representing k TRSs, and left truncation is used to account for dropouts and lowly expressed values. The expression of gene i in cell j can then be assigned to the TRS under whose Gaussian distribution the observed value is most likely to occur. All parameters in this step are estimated by MLE.
Given an input scRNA-seq expression matrix X, the feature autoencoder extracts a lower-dimensional embedding X' and reconstructs the expression $\hat{X}$, which is composed of two dense layers in both the encoder and the decoder. The loss function is MSE integrated with gene regulation information

$$\mathcal{L} = (1-\alpha)\sum(X-\hat{X})^2 + \alpha\sum\left((X-\hat{X})^2 \odot \mathrm{TRS}\right) \tag{1}$$

where $\alpha \in [0,1]$ and $\odot$ denotes element-wise product.
A KNN cell graph is constructed from the learned embeddings in the feature autoencoder, which is pruned by the Isolation Forest. With node feature matrix X', the two-layer GCN encoder is constructed as

$$Z = \mathrm{ReLU}(\tilde{A}\text{ReLU}(\tilde{A}X'W_1)W_2). \tag{2}$$

where $\tilde{A}$ is the symmetrically normalized adjacency matrix of the cell graph and W_1, W_2 are learnable parameters. The associated decoder is:

$$\hat{A} = \text{sigmoid}(ZZ^\top) \tag{3}$$

The parameters are learned through the cross-entropy loss

$$\mathcal{L}(A,\hat{A}) = -\frac{1}{N^2}\sum_{i=1}^{N}\sum_{j=1}^{N}\left(a_{ij} * \log\left(\hat{a}_{ij}\right) + (1-a_{ij}) * \log\left(1-\hat{a}_{ij}\right)\right) \tag{4}$$

where a_{ij} and $\hat{a}_{ij}$ are elements in A and $\hat{A}$.
The k-means clustering is then applied to the learned embedding in the graph autoencoder. The number of clusters is determined by the Louvain algorithm. Each cell cluster has an autoencoder to regenerate gene expressions within a cluster, whose structure is the same as the feature autoencoder without being regularized by TRS. The reconstructed gene expression is fed back into the feature encoder iteratively until the convergence of adjacency matrix ($\tilde{A}_t - \tilde{A}_{t-1} < \gamma_1$)and cell clusters converge (ARI$> \gamma_2$).
After convergence, a final imputation autoencoder is trained, which has a similar structure and loss to the feature autoencoder but with three additional regularizations:

$$\begin{aligned}\mathcal{L} = {} & (1-\alpha)\sum(X-\hat{X})^2 + \alpha\sum\left((X-\hat{X})^2 \circ \text{ TRS }\right) \\ & + \beta\sum|w| + \gamma_1\sum\left(A\cdot(X-\hat{X})^2\right) + \gamma_2\sum\left(B\cdot(X-\hat{X})^2\right)\end{aligned} \tag{5}$$

where $\beta \in [0,1]$ controls the intensity of the $L1$ penalization and B is a matrix indicating whether two cells belong to the same cluster.

dance.modules.single_modality.imputation.graphsci GraphSCI [41] is a GNN-based method to impute scRNA-seq data expressions. Given a gene expression matrix X with N genes and M cells, a gene graph associated with an $N \times N$ adjacency matrix A is derived based on gene-wise Pearson correlation coefficients. The gene expression matrix and the constructed gene graph are the input to 1) a two-layer GCN whose lower-dimensional embedding

gives the Gaussian distribution representing gene relationships and 2) a two-layer fully connected neural network (NN) from which a ZINB distribution for scRNA-seq data expressions can be inferred. In particular, the GCN is formulated as

$$H_{\mathcal{N}}^{(1)} = \text{ReLU}\left(\tilde{A}XW_{\mathcal{N}}^{(0)}\right) \tag{6}$$

$$\left[\mu_{\mathcal{N}}, \sigma_{\mathcal{N}}^2\right] = \tilde{A}H_{\mathcal{N}}^{(1)}W_{\mathcal{N}}^{(1)} \tag{7}$$

where $W_{\mathcal{N}}^{(0)}, W_{\mathcal{N}}^{(1)}$ are the trainable parameters.
The fully-connected neural network is defined as

$$H_{\mathcal{M}}^{(1)} = \tanh\left(X^{\top}\left(W_{\mathcal{M}}^{(0)} \odot A\right) + b^{(0)}\right) \tag{8}$$

$$\left[\mu_{\mathcal{M}}, \theta_{\mathcal{M}}, \pi_{\mathcal{M}}\right] = \sigma\left(H_{\mathcal{M}}^{(1)}W_{\mathcal{M}}^{(1)} + b^{(1)}\right) \tag{9}$$

where $\mu_{\mathcal{M}}, \theta_{\mathcal{M}}, \pi_{\mathcal{M}}$ are the mean, dispersion, and dropout probability for the ZINB distribution, and $W_{\mathcal{M}}^{(0)}, W_{\mathcal{M}}^{(1)}, b^{(0)}, b^{(1)}$ are trainable parameters.
The learned latent variables $\left[\mu_{\mathcal{N}}, \sigma_{\mathcal{N}}^2\right], \left[\mu_{\mathcal{M}}, \theta_{\mathcal{M}}, \pi_{\mathcal{M}}\right]$ are re-parameterized as $Z^{\mathcal{N}}$ and $Z^{\mathcal{M}}$, i.e, the latent space representation of the GCN and NN, to be used by the decoder to construct the imputed gene expression $\hat{X}$. Specifically, the imputed gene expression for gene i in cell j is

$$\hat{X}_{ij} = g_{\varphi_1}\left(Z^{cM_j}\right) = \text{diag}\left(\vec{s}_j\right) \times Z_j^{\mathcal{M}} \tag{10}$$

where $\vec{s}_j$ is the size factor of cell j.
Meanwhile, the reconstructed edge weight between gene i and gene j is

$$\hat{A}_{ij} = g_{\varphi_2}\left(Z_i^{\mathcal{N}}, Z_j^{\mathcal{N}}\right) = \text{sigmoid}\left(\left(Z_i^{\mathcal{N}}\right)^{\top} Z_j^{\mathcal{N}}\right) \tag{11}$$

$Z^{\mathcal{N}}$ and $Z^{\mathcal{M}}$ are optimized by variational lower bound

$$\begin{aligned}\mathcal{L}(\phi, \varphi) = \mathbb{E}_{q_\phi}\left[\sum_{i \in \mathcal{N}, j \in \mathcal{M}} \log p_{\varphi_1}\left(\hat{X}_{ij} \mid Z_i^{\mathcal{N}}, Z_j^{\mathcal{M}}\right)\right] + \mathbb{E}_{q_\phi}\left[\sum_{i,j \in \mathcal{N}} \log p_{\varphi_2}\left(\hat{A}_{ij} \mid Z_i^{\mathcal{N}}, Z_j^{\mathcal{N}}\right)\right] \\ - D_{KL}\left(q_\phi\left(Z^{\mathcal{M}} \mid A, X^{\top}\right) \| p\left(Z^{\mathcal{M}}\right)\right) - D_{KL}\left(q_\phi\left(Z^{\mathcal{N}} \mid A, X^{\top}\right) \| p\left(Z^{\mathcal{N}}\right)\right)\end{aligned} \tag{12}$$

where $\mathbb{E}_{q_\phi}$ is the cross-entropy function and and $D_{KL}(q||p)$ is the Kullback-Leibler divergence between distributions q and p.

A.1.2 Cell Type Annotation

dance.modules.single_modality.cell_type_annotation.scdeepsort Scdeepsort [26] includes three modules: an embedding layer, a weighted graph aggregator, and linear classification towers. Given m genes and n cells, we have the input single-cell data matrix $D \in \mathbb{R}^{m \times n}$. To generate the weighted cell-gene graph, we first apply PCA to extract d dimensional representations of initial representations. A weighted adjacency matrix $A \in \mathbb{R}^{(m+n)^2}$ and a node embedding $X \in \mathbb{R}^{(m+n) \times d}$ are generated from D. For a gene node j, the shareable parameter β_j denotes the confidence value for the edges interacting with node j. For the self-loop edge for each cell, we use α to denote its confidence value. Let h_i^k be the node i's embedding vector in the k_{th} layer, the aggregator layer is

$$h_i^k = \sigma(W^{k-1}\frac{\alpha h_i^{k-1} + \sum_{j \in N(i)} \beta_j a_{ij} h_j^{k-1}}{1 + |N(i)|} + b^{k-1}), \tag{13}$$

where a_{ij} is the normalized weight of an edge from nodes i to j. Then cell node representations are fed into linear classifier layers

$$\hat{y}_i = softmax(Wh_i^k + b) \tag{14}$$

Cross entropy loss is applied in the objective function:

$$\hat{\theta} = \text{argmin}_\theta \sum_{c=1}^{C} y_c \log \hat{y}_c \tag{15}$$

where θ represents all trainable parameters.
dance.modules.single_modality.cell_type_annotation.celltypist Celltypist [64] uses a multinomial logistic regression classifier defined as:

$$l(x) = \frac{1}{1 + e^{-w^t x}}, \tag{16}$$

where $l(x)$ is the decision score with x as the input vector. Then the decision score for each cell is defined as the linear combination of the scaled gene expression and the model coefficients associated with the given gene type, and the possibility is calculated by transforming the decision score by a sigmoid function.
dance.modules.single_modality.cell_type_annotation.singlecellnet SingleCellnet [65] revamped the random forest classifier method to enable classification of scRNA-seq data cross platforms and cross-species. It sends the input features into a number of decision tree classifiers and uses majority voting to make predictions.
dance.modules.single_modality.cell_type_annotation.actinn ACTINN [33] proposes a neural network based model for cell type annotation. It applies multilayer perceptron for the identification of cell types that can be implemented as:

$$x_i = g(W_i x_{i-1} + b_{i-1}), \tag{17}$$

where x_i is the output from the i_{th} layer, W_i and b_i represent the weight matrix and the bias in the $i_t h$ layer and g represents the activation function used in the neural network. In the input layers and hidden layers, the activation function is ReLU as:

$$ReLU(x) = max(0, x). \tag{18}$$

It utilizes the softmax function as the activation function g for the output layer, which is defined as:

$$softmax(x_j) = \frac{exp(x_j)}{\sum_{i=1}^{k} exp(x_i)} \tag{19}$$

where x_j represents the j_{th} element of the input vector for the output layer and k is the length of the vector x.
dance.modules.single_modality.cell_type_annotation.svm Support vector machine(SVM) is widely adopted as a benchmark in many studies [26, 66]. Given the input x and label y, the prediction function is

$$\hat{y} = wx + b, \tag{20}$$

where w represents the weights and b is the interception in the SVM. Based on Eq 20, SVM optimizes the following problem:

$$\min_{w,b} \frac{1}{2} ||w||^2,$$

$$subject\,to\; y(wx + b) - 1 \geq 0.$$

A.1.3 Clustering
dance.modules.single_modality.clustering.scdeepcluster scDeepCluster [34] introduces a ZINB-based autoencoder. The input matrix is corrupted by a Gaussian noise e: $X^{\text{corrupt}} = X + e$. Then the encoder produces latent representation Z from X^{corrupt}. The decoder can be formulated as:

$$\begin{aligned} \Pi &= \text{sigmoid}\left(W_\pi f_D(Z)\right) \\ M &= \text{diag}(s_i) \times \exp\left(W_\mu f_D(Z)\right) \\ \Theta &= \exp\left(W_\theta f_D(Z)\right) \end{aligned} \tag{21}$$

where $f_D(\cdot)$ is the decoder function; s_i is the size factor; $\{\Pi, M, \Theta\}$ are the estimations of ZINB distribution parameters $\{\pi, \mu, \theta\}$, respectively. The clustering process is based on soft assignment. The soft label q_{ij} of embedded point z_i is defined as:

$$q_{ij} = \frac{\left(1 + \|z_i - \mu_j\|^2\right)^{-1}}{\sum_k \left(1 + \|z_i - \mu_k\|^2\right)^{-1}} \tag{22}$$

where μ_j is the j-th cluster center.

dance.modules.single_modality.clustering.scdcc scDCC [52] shares the same model structure as scDeepCluster. In the training process, pairwise constraints are integrated into the loss function. There are two types of pairwise constraints, i.e., must-link (ML) and cannot-link (CL). Two instances with a must-link constraint should have similar soft labels as:

$$L_{\text{ML}} = -\sum_{(a,b)\in ML} \log \sum_j q_{aj} \times q_{bj} \tag{23}$$

While two instances with cannot-link should have different soft labels as:

$$L_{\text{CL}} = -\sum_{(a,b)\in CL} \log \left(\sum_j q_{aj} \times q_{bj} \right) \tag{24}$$

where q_{aj} and q_{bj} are soft labels defined in (22).

dance.modules.single_modality.clustering.graphsc graph-sc [25] utilizes gene-to-cell graph as the input of graph autoencoder. In the gene-to-cell graph, genes and cells are nodes, and there are weighted edges between cell nodes and the expressed gene nodes. Let the raw data matrix be X, then the weight of gene i to cell j is $w_{ij} = \frac{X[i,j]}{\sum_{k=0}^{m} X[k,j]}$. We use W, Z_0, and A to denote the graph weight matrix, the input features, and the adjacency matrix. $\bar{A} = D^{-\frac{1}{2}} A D^{-\frac{1}{2}}$ is the normalized adjacency matrix with D as the degree matrix of A. The cell embeddings can be obtained by:

$$Z = \text{ReLU}(\text{ReLU}(\bar{A} Z_0 W_1 W) W_2) \tag{25}$$

where W_1 and W_2 are learnable weights. The new adjacency matrix $\hat{A}$ is then reconstructed by $\hat{A} = \text{sigmoid}(ZZ^\top)$.

dance.modules.single_modality.clustering.sctag scTAG [51] first generates a K-nearest neighbor cell-to-cell graph. It then adopts a ZINB-based graph autoencoder to process it, which takes topology adaptive graph convolutional network (TAGCN) [63] as the graph encoder. Consider the l-th hidden layer, let the input data be $x_c^{(l)} \in \mathbf{R}^N$, where $c = 1, 2, \ldots, C_l$, C_l is the number of features of each node, and N denotes the number of samples. The graph convolution process can be defined as follows:

$$x_f^{(l+1)} = \text{ReLU}\left(\sum_{c=1}^{C_l} \sum_{k=0}^{K} g_{c,f,k}^{(l)} A^k x_c^{(l)} + b_f \mathbf{1}_N \right) \tag{26}$$

where b_f is a learnable bias; K is the number of convolution kernels; and $g_{c,f,k}^{(l)}$ denotes the polynomial coefficients. Denote Z as the latent embedded representation. The new adjacency matrix $\hat{A}$ is then reconstructed by $\hat{A} = \text{sigmoid}(ZZ^\top)$. The estimations of ZINB distribution parameters $\{\pi, \mu, \theta\}$ are obtained by (21).

dance.modules.single_modality.clustering.scdsc scDSC [35] consists of a ZINB-based autoencoder and a graph autoencoder with the KNN cell-to-cell graph. In the ZINB-based autoencoder, let the latent representation be H, the output of last decoding layer be D, b_{enc} and b_{dec} be bias of encoder and decoder, respectively. The autoencoder is formulated as follows:

$$\begin{aligned} H &= f_{\text{enc}}(W_{\text{enc}} X + b_{\text{enc}}) \\ \bar{X} &= f_{\text{dec}}(W_{\text{dec}} H + b_{\text{dec}}) \end{aligned} \tag{27}$$

where $\bar{X}$ is the reconstructed expression matrix. The estimations of ZINB distribution parameters $\{\pi, \mu, \theta\}$ are similar to those in (21). In the graph encoder, denote the representation of l-th layer as $Z_{(l)}$, the adjacency matrix as A, and the degree matrix as D. The new representation is generated by:

$$Z_{(l+1)} = \phi\left(D^{-\frac{1}{2}} A D^{-\frac{1}{2}} \left(\sigma Z_{(l)} + (1-\sigma) H_{(l)} \right) W_{(l)} \right) \tag{28}$$

where $\phi(\cdot)$ is an activation function; σ is a hyperparameter; and $H_{(l)}$ is the representation of l-th layer of ZINB-based encoder.

A.2 Multimodality Module

A.2.1 Modality Prediction

dance.modules.multi_modality.predict_modality.scmogcn scMoGNN [11] first converts the input feature matrix into a cell-feature bipartite graph $\mathcal{G} = (\mathcal{V}, \mathcal{E})$ where each node v represents a cell or a feature. For instance, for the input of gene expression, each node can be a cell or a gene. Meanwhile, additional gene-gene connections are added based on an external pathway dataset [92]. Every pair of vertices in V are connected by a weighted edge, which either depends on the read count of a feature in a cell, or the correlation between features.
Specifically, we use $\mathbf{X} \in \mathbb{R}^{N \times k}$ to denote the feature matrix of input modality with N the number of cells and k the dimension of input features. The adjacency matrix $\mathbf{A}$ of the constructed cell-feature bipartite graph $\mathcal{G}$ is:

$$\mathbf{A} = \begin{pmatrix} \mathbf{O} & \mathbf{X} \\ \mathbf{X}^T & \mathbf{P} \end{pmatrix}, \tag{29}$$

where $\mathbf{O}$ is a zero matrix, and $\mathbf{P} \in \mathbb{R}^{k \times k}$ indicates the gene-gene links.
To learn node embeddings on $\mathcal{G}$, scMoGNN introduce a heterogeneous graph convolutional network. The proposed heterogeneous graph convolutional network can be stated as:

$$\mathbf{H}_{\mathsf{c}}^{(l+1)} = \mathsf{GCN}_{\mathsf{fc}}(\mathbf{A}_{\mathsf{fc}}, \mathbf{H}^{(l)}), \tag{30}$$

$$\mathbf{H}_{\mathsf{f}}^{(l+1)} = \mathsf{GCN}_{\mathsf{cf}}(\mathbf{A}_{\mathsf{cf}}, \mathbf{H}^{(l)}) + \mathsf{GCN}_{\mathsf{fc}}(\mathbf{A}_{\mathsf{ff}}, \mathbf{H}^{(l)}), \tag{31}$$

where $\mathbf{H}_{\mathsf{f}}^{(l)}$ is the embeddings of feature nodes in l-th layer, $\mathbf{H}_{\mathsf{c}}^{(l)}$ is the embeddings of cell nodes in l-th layer. Subscripts cf, $textfc$ and $textff$ denote the graph convolution over all the cell-to-feature edges, feature-to-cell edges and feature-to-feature edges respectively. After stacking convolutional layers, cell node embeddings from each layer are collected by a weighted sum, denoted as

$$\hat{\mathbf{H}} = \sum_{l=1}^{L} \mathbf{w}_l \mathbf{H}_c^l, \tag{32}$$

where $\mathbf{w}$ is a learnable weight vector, L is the total number of layers. $\hat{\mathbf{H}}$ is then passed to the downstream modules. In the case of modality prediction, a fully-connected predictive head is added. The final prediction $\mathbf{Z} \in \mathbb{R}^{N \times d}$ of scMoGNN can be thus written as:

$$\mathbf{Z} = \mathsf{ReLU}\left(\hat{\mathbf{H}}\mathbf{W} + \mathbf{b}\right) \tag{33}$$

where $\mathbf{W}$ and $\mathbf{b}$ are the parameters of the predictive head. Overall, a mean squared error $\mathsf{MSE}(\mathbf{Z}, \mathbf{Y})$ is optimized through training, where $\mathbf{Y} \in \mathbb{R}^{N \times d}$ is ground-truth features of the target modality.

dance.modules.multi_modality.predict_modality.babel BABEL [36] trains two neural-network-based encoders and two decoders on the paired data to translate data from one modality to the other and to reconstruct itself, thus eventually obtaining shared embedding. A special design in BABEL is to add a prior distribution to the decoder. Instead of directly outputting the feature values, the decoder estimates the parameters of feature distribution. Formally, for each cell, the RNA decoder models the likelihood of expressions y as a negative binomial (NB) distribution, written as:

$$P(y; \hat{y}, \theta) = \frac{\Gamma(y+\theta)}{y!\Gamma(y)} \left(\frac{\theta}{\theta + \hat{y}}\right)^{\theta} \left(\frac{\hat{y}}{\theta + \hat{y}}\right)^{y} \tag{34}$$

where Γ denotes the gamma function, $\hat{y}$ and θ are the estimation of the mean and dispersion of the distribution. In practice, these estimations come from neural network encoders and decoders. The optimization problem is thus formalized by minimizing the negative log-likelihood:

$$\begin{aligned} \mathcal{L}_{\mathsf{NB}}(y; \hat{y}, \theta) = & -\theta(\log(\theta+\epsilon) - \log(\theta+\hat{y})) - y(\log(\hat{y}+\epsilon) - \log(\theta+\hat{y})) \\ & - \log\Gamma(y+\theta) + \log\Gamma(y+1) + \log\Gamma(\theta+\epsilon) \end{aligned} \tag{35}$$

where ϵ is a tiny constant for numerical stability.
The ATAC decoder has a different modeling approach since the feature for each peak is binary. The loss function for the ATAC decoder is based on binary cross-entropy, shown below:

$$\mathcal{L}_{\mathsf{BCE}}(x;\widehat{x}) = -(x\log\widehat{x} + (1-x)\log(1-\widehat{x})) \tag{36}$$

where x represents ground-truth ATAC-seq features, and $\widehat{x}$ denotes the prediction from the ATAC decoder.
The overall loss function is hereby formulated as:

$$\mathcal{L} = \mathcal{L}_{\mathsf{NB}}\left(r, r_{\mathsf{RNA}}\right) + \beta\mathcal{L}_{\mathsf{BCE}}\left(a, a_{\mathsf{ATAC}}\right) + \beta\mathcal{L}_{\mathsf{BCE}}\left(a, a_{\mathsf{RNA}}\right) + \mathcal{L}_{\mathsf{NB}}\left(r, r_{\mathsf{ATAC}}\right) \tag{37}$$

where r and a are the ground-truth RNA-seq features and ATAC-seq features respectively, subscripts indicate from which modality the features are predicted (e.g., a_{RNA} represents the ATAC features predicted from RNA). The first two terms in the loss function are similar to reconstruction loss in autoencoders. The latter two terms can be considered as modality prediction loss.
For testing, we simply take a_{RNA} as the prediction of ATAC-seq from RNA-seq, and take r_{ATAC} as the prediction of RNA-seq from ATAC-seq.

dance.modules.multi_modality.predict_modality.cmae Cross-modal Autoencoders [37] uses autoencoders to map vastly different modalities (including images) to a shared latent space. Specifically, a discriminator and adversarial loss are added to force the distributions of different modalities to be matched in the latent space. To make use of prior knowledge, an additional loss term can further be added to align specific markers or anchoring cells.
Formally, an invariant latent distribution for two modalities i and j is learned as follows. We denote the input features of two modalities as $\mathbf{X}_i$ and $\mathbf{X}_j$. For modality i, we optimize the objective:

$$\min_{\mathsf{E}_i,\mathsf{D}_i} \mathbb{E}_{\mathbf{x}\sim P_{\mathbf{X}_i}} \mathcal{L}_1\left(\mathbf{x}, \mathsf{D}_i\left(\mathsf{E}_i(\mathbf{x})\right)\right) + \lambda\mathcal{L}_2\left(\mathsf{E}_i \# P_{\mathbf{X}_i} \mid P_{\hat{\mathbf{Z}}}\right) \tag{38}$$

while for modality j, we optimize:

$$\min_{\mathsf{E}_j,\mathsf{D}_j} \mathbb{E}_{\mathbf{x}\sim P_{\mathbf{X}_j}} \mathcal{L}_1\left(\mathbf{x}, \mathsf{D}_j\left(\mathsf{E}_j(\mathbf{x})\right)\right) + \lambda\mathcal{L}_2\left(\mathsf{E}_j \# P_{\mathbf{X}_j} \mid P_{\hat{\mathbf{Z}}}\right) \tag{39}$$

Here, $\mathsf{E}_i \# P_{\mathbf{X}_i}$ refers to the distribution of modality i in the latent space Z, $P_{\hat{\mathbf{Z}}_i}$ is the expected distribution of joint latent space Z. D and E refer to encoders and decoders parameterized by neural networks. $\mathcal{L}_1$ is the Euclidean distance metric, which is equivalent to a reconstruction loss. $\mathcal{L}_2$ represents a divergence between probability distributions, since $P_{\hat{\mathbf{Z}}_i}$ is unknown, it can be adapted to a discriminator and an adversarial loss.
Several additional losses can be added to the model to incorporate prior knowledge. For example, if training data include paired multimodal data, those cells with more than one modality can be considered as anchors. Suppose $\left(x_1, x_1'\right), \left(x_2, x_2'\right), \ldots, \left(x_m, x_m'\right)$ are corresponding points from two datasets, we can add the following anchor loss,

$$\sum_{i=1}^{m} ||\mathsf{E}\left(x_i\right) - \mathsf{E}'\left(x_i'\right)|| \tag{40}$$

where E and E$'$ are encoders for the two modalities, respectively.
The final prediction from modality i to modality j is $\mathsf{D}_j\left(\mathsf{E}_i(\mathbf{X})\right)$.

dance.modules.multi_modality.predict_modality.scmm scMM [38] leverages a mixture-of-experts (MoE) multimodal variational autoencoder [87] (VAE) to explore the latent dimensions that associate with multimodal regulatory programs. It models raw count features from each modality using various probability distributions in an end-to-end way. Specifically, an MoE multimodal VAE (MMVAE) is to learn a multimodal generative model:

$$p_\Theta\left(\boldsymbol{z}, \boldsymbol{x}_{1:M}\right) = p(\boldsymbol{z}) \prod_{m}^{M} p_{\theta_m}\left(\boldsymbol{x}_m \mid \boldsymbol{z}\right), \tag{41}$$

where $p_{\theta_m}\left(\boldsymbol{x}_m \mid \boldsymbol{z}\right)$ is the likelihood for m-th modality, and p_{θ_m} is parameterized by a neural network decoder.
To optimize the model, a typical training objective for VAE is to maximize the ELBO:

$$\mathsf{ELBO} = \mathbb{E}_{z\sim q_\Phi}\left(z \mid \boldsymbol{x}_{1:M}\right)\left[\log \frac{p_\Theta\left(z, x_{1:M}\right)}{q_\Phi\left(z \mid x_{1:M}\right)}\right] \tag{42}$$

where $q_\Phi\left(\boldsymbol{z} \mid \boldsymbol{x}_{1:M}\right)$ is the joint variational posterior that can be parameterized by a neural network encoder. In addition, an MMVAE factorizes the joint posterior with an MoE:

$$q_\Phi\left(\boldsymbol{z} \mid \boldsymbol{x}_{1:M}\right) = \sum_{m}^{M} \alpha_m q_{\varphi_m}\left(\boldsymbol{z} \mid \boldsymbol{x}_m\right), \alpha_m = 1/M, \tag{43}$$

where the posterior of the m-th modality is $q_{\varphi_m}(\boldsymbol{z} \mid \boldsymbol{x}_m)$. It is parameterized by the encoder. When using stratified sampling, ELBO can be re-written as:

$$\begin{aligned} \text{ELBO} &= \frac{1}{M}\sum_{m}^{M}\mathbb{E}_{\boldsymbol{z}_m \sim q_{\varphi_m}}(z \mid \boldsymbol{x}_m)\left[\log\frac{p_\Theta(z_m, \boldsymbol{x}_{1:M})}{q_{\Phi}(\boldsymbol{z}_m \mid \boldsymbol{x}_{1:M})}\right] \\ &= \frac{1}{M}\sum_{m}^{M}\left\{\mathbb{E}_{\boldsymbol{z}_m \sim q_{\varphi_m}(\boldsymbol{z} \mid \boldsymbol{x}_{m_i})}\left[\log p_\Theta(\boldsymbol{x}_{1:M} \mid \boldsymbol{z}_m)\right] - \text{KL}\left[q_{\varphi_m}(\boldsymbol{z} \mid \boldsymbol{x}_m)\,\|p(\boldsymbol{z})\right]\right\} \end{aligned} \tag{44}$$

The first expectation term is to measure the reconstruction performance. Here, latent variables of each modality would be used to reconstruct all modalities, including cross-modal translation. The second term is a regularization term forcing the variational posterior to be consistent the prior distribution $p(\boldsymbol{z})$, which is a Laplacian distribution in practice. In the modality prediction task, we take the cross-modal generation results as the prediction of the model.

A.2.2 Modality Matching

dance.modules.multi_modality.match_modality.scmogcn The overall structure of scMoGNN in the modality matching task is the same as in the modality prediction task. However, in the modality prediction task, the input is only one modality, while in the modality matching task, features of two modalities are given altogether. Therefore, scMoGMM constructs two graphs $\mathcal{G}_1$ and $\mathcal{G}_2$ for two modalities respectively. The cell node embeddings are obtained in the same way as before, denoted as $\hat{\mathbf{H}}_1$ and $\hat{\mathbf{H}}_2$. Then a matching head is added, formulated as:

$$\mathbf{S} = \hat{\mathbf{H}}_1 \cdot \hat{\mathbf{H}}_2^T \tag{45}$$

where $\mathbf{S}$ is the desired output matrix of matching scores. For training, we calculate the cross entropy loss between $\mathbf{S}$ and a ground-truth matching matrix $\mathbf{M} \in \mathbb{R}^{n \times n}$, where $\mathbf{M}_{i,j} = 1$ only when cell i in modality 1 corresponds to cell j in modality 2. In addition, several auxiliary losses are added, including a reconstruction loss and a translation loss.

For testing, a bipartite matching via the Hungarian algorithm is implemented as post-processing. It generates an optimized sparse score matrix over the raw $\mathbf{S}$ matrix.

dance.modules.multi_modality.match_modality.cmae The overall structure of Cross-modal Autoencoders is the same as in the modality prediction task, where we implement encoders and decoders for all the modalities. Hereby in the modality matching task, we directly utilize the latent space instead of using a decoder to generate target modality.

To be specific, the embeddings from each modality can be denoted as:

$$\mathbf{H}_1 = \mathsf{E}_1(\mathbf{X}_1), \mathbf{H}_2 = \mathsf{E}_2(\mathbf{X}_2) \tag{46}$$

where we denote input matrix, encoder and embeddings of m-th modality as E_m, $\mathbf{H}_m$ and $\mathbf{X}_m$ respectively. Then the score matrix is obtained by:

$$\mathbf{S} = \mathbf{H}_1 \cdot \mathbf{H}_2^T \tag{47}$$

where $\mathbf{S}$ is the output score matrix.

dance.modules.multi_modality.match_modality.scmm The overall structure of scMM is the same as in the modality prediction task, where we implemented a neural network encoder E_m for each modality m to estimate the variational posterior $q_\Phi(\boldsymbol{z} \mid \boldsymbol{x}_{1:M})$. In the modality matching task, we hereby take the latent vectors generated by encoders as the source for matching. The whole process is the same as Eq. 46 and 47.

A.2.3 Joint Embedding

dance.modules.multi_modality.joint_embedding.scmogcn The overall structure of scMoGNN in the joint embedding task is still similar to what is shown in the modality prediction task. However, different from previous tasks, here scMoGNN first preprocesses data as suggested by Seurat [93]. It reduces the dimension of modality m to a predefined k_m dimension, using latent semantic indexing (LSI). Empirically, we set $k_m = 256$ for RNA-seq features, $k_m = 512$ for ATAC-seq features, and no dimension reduction for surface protein features. Next, the preprocessed features of two modalities are concatenated and jointly considered as feature nodes in the graph construction, as described in Eq. 29. Since no feature interactions are specified, here we replace matrix $\mathbf{P}$ in Eq. 29 with $\mathbf{O}$. With the graph $\mathcal{G}$ so constructed, scMoGNN is further trained by minimizing a reconstruction loss, a cell type auxiliary loss and a regularization loss.

Specifically, cell embeddings $\hat{\mathbf{H}}$ are obtained as Eq. 32. An MLP decoder f_θ is involved to reconstruct the input features from $\hat{\mathbf{H}}$. The first T dimensions in $\hat{\mathbf{H}}$ are also used to predict cell type, where T is equal to the number of predefined cell types, and a regularization loss is added to the rest of the dimensions. The overall loss function can

be written as:

$$
\begin{aligned}
\mathcal{L} &= \mathcal{L}_{\text{recon}} + \mathcal{L}_{\text{cell type}} + \mathcal{L}_{\text{regular}} \\
&= \frac{1}{N}\sum_{i=1}^{N}(\mathbf{X} - f_\theta(\hat{\mathbf{H}}))^2 + \sum_{t=1}^{T}\mathbf{Y}_t \log(\hat{\mathbf{Y}}_t) + \beta * \|\hat{\mathbf{H}}_{\bar{\mathcal{J}}}\|_2
\end{aligned} \tag{48}
$$

where f_θ is a two-layer MLP decoder, $\mathbf{X} \in \mathbb{R}^{n\times(k_1+k_2)}$ is the pre-processed feature matrix, k_1 and k_2 are specified feature dimensions of two modalities, $\mathbf{Y} \in \mathbb{R}^{N\times T}$ is predefined cell types for each cell in a sparse form, and $\hat{\mathbf{H}}_{\bar{\mathcal{J}}}$ refers to the hidden dimensions other than first T dimensions. $\hat{\mathbf{Y}}$ is calculated by a softmax function over the first T dimensions of $\hat{\mathbf{H}}$, formulated as:

$$
\hat{\mathbf{Y}}_{i,t} = \frac{e^{\hat{\mathbf{H}}_{i,t}}}{\sum_{k=1}^{T} e^{\hat{\mathbf{H}}_{i,k}}} \tag{49}
$$

In the end, $\hat{\mathbf{H}}$ is the resulting joint cell embeddings from scMoGNN, which is expected to encode cellular information that is essential in the joint embedding task.

dance.modules.multi_modality.joint_embedding.jae JAE is an adapted model from scDEC [72]. It is proposed by the authors of scDEC in the NeurIPS competition [84] to better leverage cell annotations. Formally, JAE follows the typical autoencoder architecture with an encoder f_θ and a decoder g_θ. They are parameterized by MLPs, denoted as:

$$
\mathbf{H} = f_\theta(\mathbf{X}), \mathbf{Z} = g_\theta(\mathbf{H}) \tag{50}
$$

where $\mathbf{H} \in \mathbb{R}^{n\times d}$ is the joint embeddings, and $\mathbf{Z}$ is the recovered input features. The novelty of JAE is that it separates cell embeddings into four parts, written as:

$$
\mathbf{H} = [\mathbf{H}'|\mathbf{C}|\mathbf{B}|\mathbf{S}] \tag{51}
$$

where $|$ denotes concatenation, $\mathbf{C} \in \mathbb{R}^{n\times c}$ is additionally supervised by cell type labels, $\mathbf{B} \in \mathbb{R}^{n\times b}$ is additionally supervised by batch labels, $\mathbf{S} \in \mathbb{R}^{n\times s}$ is additionally supervised by cell cycle phase score, $\mathbf{H}' \in \mathbb{R}^{n\times z}$ is the remaining dimensions. Therefore $z + b + s + c = d$. The overall loss function of JAE is formulated as:

$$
\begin{aligned}
\mathcal{L} &= \mathcal{L}_{\text{recon}} + \mathcal{L}_{\text{cell\_type}} + \mathcal{L}_{\text{batch}} + \mathcal{L}_{\text{cell\_cycle}} \\
&= \text{MSE}(\mathbf{Z}, \mathbf{X}) + \text{CrossEntropy}(\mathbf{C}, \hat{\mathbf{C}}) \\
&\quad + \text{CrossEntropy}(\mathbf{B}, \hat{\mathbf{B}}) + \text{MSE}(\mathbf{SW} + \mathbf{b}, \hat{\mathbf{S}})
\end{aligned} \tag{52}
$$

where $\hat{\mathbf{C}}$, $\hat{\mathbf{B}}$, $\hat{\mathbf{S}}$ are cell type labels, batch labels and cell cycle scores, respectively. $\mathbf{W} \in \mathbb{R}^{s\times 2}$ and $\mathbf{b} \in \mathbb{R}^2$ are an extra linear transformation to project $\mathbf{S}$ to cell cycle score vector space. Eventually, $\mathbf{H}$ is the output joint embedding from JAE.

dance.modules.multi_modality.joint_embedding.scmvae The scMVAE [39] model employs three simultaneous learning strategies to understand the distribution of multi-omics data: the product of experts (PoE), neural networks, and concatenation of multi-omics features. Furthermore, scMVAE handles raw count features from each modality using a Zero-Inflated Negative Binomial (ZINB) distribution. Specifically, let z represent the joint embeddings obtained from multimodal encoders. The conditional distribution $p(z \mid c)$ follows a Gaussian mixture distribution, with its mean vector μ_c and covariance matrix σ_c conditioned on a discrete categorical variable c indicating cell types. This Gaussian mixture prior for z is introduced in MVAE to improve the interpretability of latent representations and enhance generation performance. Considering x and y as features of two modalities, the distribution $p(x, y, z, c, l_x, l_y)$ is formulated as follows:

$$
p(x, y, z, c, l_x, l_y) = p(x \mid z, l_x)\, p(y \mid z, l_y)\, p(z \mid c) p(c) p(l_x)\, p(l_y) \tag{53}
$$

where l_x and l_y are the library size factors of two modalities.

The prior distributions for all the random variables are listed below:

$$
\begin{aligned}
& z \sim N\left(\mu_c, \sigma_c^2 I\right) \\
& l_x \sim \log \operatorname{norm}\left(\mu_{l x}, \sigma_{l x}^2\right), l_y \sim \log \operatorname{norm}\left(\mu_{l y}, \sigma_{l y}{ }^2\right) \\
& \mu_x \sim \operatorname{Gamma}\left(f_{\mu x}(f(z)), f_{\theta x}(f(z))\right), \mu_y \sim \operatorname{Gamma}\left(f_{\mu y}(f(z)), f_{\theta y}(f(z))\right) \\
& x^{\prime} \sim \text { Possion }\left(l_x \mu_x\right), y^{\prime} \sim \text { Possion }\left(l_y \mu_y\right) \\
& \pi_x \sim \operatorname{Bernoulli}\left(f_{\pi x}(f(z))\right), \pi_y \sim \operatorname{Bernoulli}\left(f_{\pi y}(f(z))\right) \\
& x_r=\left\{\begin{array}{l}
x^{\prime} \text { if } \pi_x=0 \\
0 \text { otherwise }
\end{array}, y_r=\left\{\begin{array}{l}
y^{\prime} \text { if } \pi_y=0 \\
0 \text { otherwise }
\end{array}\right.\right.
\end{aligned} \tag{54}
$$

where $f_{\theta x}\left(f(z)\right)$ and $f_{\theta y}\left(f(z)\right)$ are the inverse desperations of two modalities from the variational Bayesian inference. $f_{\mu x}$ and $f_{\mu y}$ are two neural network decoders that estimate the mean proportions of features for two modalities in each cell by using a softmax function, which simulates the library-size normalized features. $f_{\pi x}$ and $f_{\pi y}$ are neural network decoders that estimate the probability of features being dropped out due to technical issues. They use a sigmoid function to model this probability.

To train scMVAE, we maximize the log-likelihood of the multi-omics observations. Following the convention of variational autoencoders, this objective is converted to optimizing an evidence lower bound (ELBO):

$$
\begin{gathered}
\log p\left(x, y \mid z, c, l_x, l_y\right) \geq E_{q_{\varphi}}\left(z, c, lx, ly \mid x, y)\right) \\
{\left[\lambda_1 \log \left(p_{\theta_1}\left(x \mid z, l_x\right)\right)+\lambda_2 \log \left(p_{\theta_2}\left(y \mid z, l_y\right)\right)\right]} \\
-\alpha_1 D_{K L}\left(q\left(l_x \mid x\right) \| p\left(l_x\right)\right)-\alpha_2 D_{K L}\left(q\left(l_y \mid y\right) \| p\left(l_y\right)\right) \\
-\beta D_{K L}(q(z, c \mid x, y) \| p(z, c))
\end{gathered} \tag{55}
$$

Both modalities in the ELBO have two reconstruction terms, and three regularization terms are implemented by KL divergence. q_{φ} refers to a multimodal encoder. While p_{θ_1} and p_{θ_2} refer to decoders, for two modalities respectively. The latent vector z estimated from E is the eventual joint embedding.

dance.modules.multi_modality.joint_embedding.dcca In DCCA [39], data of each modality are modeled by a variational autoencoder (VAE). Specifically, for modality m, an encoder E_m transforms the input features into latent space z_m. A decoder D_m then transforms z_m into the parameters of the NB or Bernoulli. For example, RNA-seq and ADT data follow NB distribution, denoted as:

$$
\begin{gathered}
p\left(x \mid z_x\right)=\mathrm{NB}\left(x ; u_x, \theta_x\right)=\mathrm{NB}\left(x, l_x ; D_{u_x}\left(z_x\right), D_{\theta_x}\left(z_x\right)\right) \\
\mathrm{NB}\left(x ; u_x, \theta_x\right)=\frac{\Gamma\left(x+\theta_x\right)}{\Gamma\left(\theta_x\right) \Gamma(x+1)}\left(\frac{u_x}{u_x+\theta_x}\right)^x\left(\frac{\theta_x}{\theta_x+u_x}\right)^{\theta_x}
\end{gathered} \tag{56}
$$

where D_{u_x} and D_{θ_x} are decoders, each dimension of u_x and θ_x indicates the mean and variance of NB distribution for each feature, and one-dimensional constant variable l_x indicates the library size of each cell. While ATAC-seq data follow Bernoulli distribution, denoted as:

$$
\begin{gathered}
p\left(y \mid z_y\right)=\operatorname{Bernoulli}\left(y ; u_y\right)=\operatorname{Bernoulli}\left(y ; D_{u_y}\left(z_y\right)\right) \\
\operatorname{Bernoulli}\left(y ; u_y\right)=y \log \left(u_y\right)+(1-y) \log \left(1-u_y\right)
\end{gathered} \tag{57}
$$

where D_{uy} refers to the ATAC-seq decoder.

Each VAE is first trained separately with each modality. Then, two VAEs are trained together to maximize the similarity between two latent spaces. For example, given the embeddings from a $\mathrm{VAE}_{\mathrm{RNA}}$ and a $\mathrm{VAE}_{\mathrm{ATAC}}$, they optimize an objective function that combines reconstruction loss with the cell embeddings similarity loss. Hence, the total ELBO for $\mathrm{VAE}_{\mathrm{RNA}}$ and $\mathrm{VAE}_{\mathrm{ATAC}}$ can be written as:

$$
\begin{gathered}
\mathcal{L}_{\mathrm{RNA}}=\log p\left(x \mid z_x\right)-\beta_1 \sum_{i=0}^{K-1}\left\|{z_y}^i-{z_x}^i\right\|_2 \\
\geq E_{z_x \sim q\left(z_x \mid x ; E_x\right)}\left(\log p\left(x \mid z_x ; D_{ux}, D_{\theta x}\right)\right)-\lambda_1 D_{K L}\left(q\left(z_x \mid x ; E_x\right) \| p(z)\right) \\
-\beta_1 \sum_{i=0}^{K-1}\left\|{z_y}^i-{z_x}^i\right\|_2
\end{gathered} \tag{58}
$$

$$\mathcal{L}_{\mathsf{ATAC}} = \log p\left(y \mid z_y\right) - \beta_2 \sum_{i=0}^{K-1} \left\| {z_x}^i - {z_y}^i \right\|_2$$
$$\geq E_{z_y \sim q\left(z_y \mid y; E_y\right)} \left(\log p\left(y \mid z_y; D_{uy}\right)\right) - \lambda_2 D_{KL}\left(q\left(z_y \mid y; E_y\right) \| p(z)\right) \tag{59}$$
$$-\beta_2 \sum_{i=0}^{K-1} \left\| {z_x}^i - {z_y}^i \right\|_2$$

According to the DCCA paper, after jointly training two VAEs, the embedding from $\mathsf{VAE}_{\mathsf{RNA}}$ is selected as the final embedding for downstream analysis.

A.3 Spatial Transcriptomics Module

A.3.1 Spatial Domain

dance.modules.spatial.spatial_domain.spagcn SpaGCN [42] first constructs a weighted undirected graph, $G(V, E)$ from the gene expression and histological image data. In G, each vertex $v \in V$ is a spot, and every pair of vertices in V are connected by a weighted edge, which assesses the correlation between the two spots. The weight for the pair $(u$,$v)$ is calculated as:

$$w(u, v) = \exp\left(-\frac{d(u, v)^2}{2l^2}\right) \tag{60}$$

where hyperparameter l represents characteristic length scale, and $d(u, v)$ calculates Euclidean distance between spots u and v. This distance is computed by the spatial distance and the corresponding histology information between two spots. The initial node representation in the graph is gene expression after dimension reduction. A process known as graph convolution is utilized by SpaGCN to aggregate gene expression data in accordance with edge weights. Then the output of the graph convolution layer would be new node representation capturing information on gene expression, histology and physical location. Based on the new spot representation, an unsupervised clustering algorithm is further employed to iteratively cluster the spots into spatial domains. The probability of assigning spot i to cluster j is defined as:

$$q_{ij} = \frac{\left(1 + h_i - \mu_j^2\right)^{-1}}{\sum_{j'=1}^{K} \left(1 + h_i - \mu_{j'}^2\right)^{-1}} \tag{61}$$

where h_i is the embedded point for spot i, and u_j indicates centroid j. Then the clusters are refined iteratively by a target distribution P from q_{ij}:

$$p_{ij} = \frac{q_{ij}^2 / \sum_{i=1}^{N} q_{ij}}{\sum_{j'=1}^{K} \left(q_{ij'}^2 / \sum_{i=1}^{N} q_{ij'}\right)} \tag{62}$$

which gives more weight to locations that have been recognized with high confidence and normalizes each centroid's contribution to the loss function to avoid having large clusters distort the hidden feature space. The objective function is based on a Kullback–Leibler (KL) divergence as:

$$L = KL(P \| Q) = \sum_{i=1}^{N} \sum_{j=1}^{K} p_{ij} \log \frac{p_{ij}}{q_{ij}} \tag{63}$$

where N is the number of spot samples, and K is the number of clusterings.

dance.modules.spatial.spatial_domain.stagate STAGATE [43] is a graph attention based autoencoder [45] with encoder, decoder and graph attention layers. For graph construction, it builds up an undirected graph with a radius r that has been predefined based on physical distance. We use$\mathbf{A}$ to denote the adjacency matrix of the graph where $\mathbf{A_{ij}} = 1$ if the Euclidean distance between spots i and j is less than r. In addition, STAGATE also builds up a cell type-aware graph via updating the previously constructed graph based on the pre-clustering of gene expressions. The encoder in STAGATE takes the gene expressions that have been normalized as its inputs. It then generates embedding for each spot by aggregating information from its surrounding nodes collectively. The latent representation for spot i is calculated as:

$$\mathbf{h}_i^{(k)} = \sigma\left(\sum_{j \in S_i} \mathbf{att}_{ij}^{(k)} \left(\mathbf{W}_k \mathbf{h}_j^{(k-1)}\right)\right) \tag{64}$$

where σ is the activation function, $\mathbf{W}_k$ is the trainable weight matrix, S_i is the neighbors of spot i, k indicates k-th encoder layer and $\mathbf{att}_{ij}^{(k)}$ is the attention score (i.e., the edge weight) between spots i and j obtained from the k-th graph attention layer's output.
Contrarily, using the encoder's output as input, the decoder transforms the latent embedding into a reconstructed normalized expression profile.

$$\widehat{\mathbf{h}}_i^{(k-1)} = \sigma\left(\sum_{j\in S_i} \widehat{\mathbf{att}}_{ij}^{(k-1)}\left(\widehat{\mathbf{W}}_k \widehat{\mathbf{h}}_j^{(k)}\right)\right) \tag{65}$$

In graph attention layer, a widely-used self-attention technique for graph neural networks is adopted to learn the similarity between surrounding spots in an adaptive manner. The edge weight between spot i and its surrounding spot j in the k-th encoder layer is calculated as:

$$e_{ij}^{(k)} = \mathrm{Sigmoid}\left(\mathbf{v}_s^{(k)T}\left(\mathbf{W}_k \mathbf{h}_i^{(k-1)}\right) + \mathbf{v}_r^{(k)T}\left(\mathbf{W}_k \mathbf{h}_j^{(k-1)}\right)\right) \tag{66}$$

where $\mathbf{v}_s^{(k)}$ and $\mathbf{v}_r^{(k)}$ are the trainable weights.
The attention score is further normalized by a softmax function in the following way:

$$att_{ij}^{(k)} = \frac{\exp\left(e_{ij}^{(k)}\right)}{\sum_{i\in\mathscr{S}_i}\exp\left(e_{ij}^{(k)}\right)} \tag{67}$$

Eventually, STAGATE aims to minimize the reconstruction loss of normalized expressions in the following way:

$$\sum_{i=1}^{N}\left\|\mathbf{x}_i - \widehat{\mathbf{h}}_i\right\|_2 \tag{68}$$

where $\mathbf{x}_i$ is the original normalized gene expressions, and $\widehat{\mathbf{h}}_i$ is the reconstructed normalized gene expression for spot i.

dance.modules.spatial.spatial_domain.louvain Louvain [73] is to extract the community structure of large networks, which draws inspiration from the optimization of modularity. A modularity measure shows how densely edges inside communities are clustered in comparison to edges outside communities. Theoretically, optimizing this value leads to the optimal grouping of nodes in a particular network. Due to the impracticality of traversing all possible iterations of the nodes into groups, heuristic approaches are utilized. The modularity is defined as:

$$Q = \frac{1}{2m}\sum_{ij}\left[A_{ij} - \frac{k_i k_j}{2m}\right]\delta\left(c_i, c_j\right) \tag{69}$$

where A_{ij} is the edge weight between nodes i and j, m represents the total weight of all edges in the graph, the weights of the edges that connect nodes i and j are added up to get k_i and k_j, c_i and c_j denote node communities, and δ represents Kronecker delta function [94] ($\delta(x, y) = 1$ if $x = y$; else, 0).
To efficiently maximize the modularity, there are two looping phases. First, a community is associated with each node in the network. Because of this primary partitioning, there is consequently the same number of communities as nodes. Then, the change in modularity is determined for each node i by removing it from its own community and inserting it in the community of each of i's neighbors j. Integration of a previously isolated node i into a community C results in an increase in modularity ΔQ equal to:

$$\Delta Q = \left[\frac{\Sigma_{in} + 2k_{i,in}}{2m} - \left(\frac{\Sigma_{tot} + k_i}{2m}\right)^2\right] - \left[\frac{\Sigma_{in}}{2m} - \left(\frac{\Sigma_{tot}}{2m}\right)^2 - \left(\frac{k_i}{2m}\right)^2\right] \tag{70}$$

where $\sum_{tot}$ is the total link weights occurring to nodes in C, $\sum_{in}$ represents the total link weights within C, k_i denotes the sum of the link weights associated with node i, $k_{i,in}$ represents the total link weights from node i to all other nodes in C, and m is the total link weights for the whole network. Once this value is computed for all communities to which i belongs, i is assigned to the community where the modularity increase was largest. If there is no possibility of expansion, i stays in the same community. This technique is repeated and applied successively to all nodes until no further growth in modularity is possible. The first step concludes when this modularity maximum is reached.
In the second phase, all nodes inside the same community are grouped together and a new network consisting of communities from the first phase is constructed. The connections between multiple nodes within the same community and a node in a separate community are now described by weighted edges between communities. The second stage is complete when the new network is set up, at which point the first stage can be applied to it again.

dance.modules.spatial.spatial_domain.stlearn stLearn [74] performs unsupervised clustering on SME-normalized data to group similar areas into clusters and discover sub-clustering alternatives based on the geographic separation of clusters inside the tissue. The name of this stLearn function is SMEcluster. Using normalized expression values, stLearn separates cell types in a tissue through a two-step spatial clustering approach. stLearn implements a conventional Louvain clustering technique for scRNAseq data as the initial step. Linear Principal Component Analysis (PCA) is used to reduce the dimensionality of the SME normalized matrix, and then non-linear UMAP embedding is used to generate the k-nearest neighbor (kNN) graph. kNN's graph adjacency matrix is then clustered using Louvain clustering or k-means clustering. In the second stage, spatial information is utilized to identify sub-clusters from large clusters that span two or more physically distinct places. Using each location's spatial coordinates, a two-dimensional k-d tree neighbour search is conducted.

A.3.2 Cell Type Deconvolution

dance.modules.spatial.cell_type_deconvo.dstg DSTG [44] is a GNN based method that constructs a graph from similarities between real mixed-cell expression data $X \in \mathbb{R}^{d\times n}$ and pseudo mixed-cell expression data $\widetilde{X} \in \mathbb{R}^{d\times n_p}$ from reference scRNA-seq expression data $X_s \in \mathbb{R}^{d\times N}$. First, the pseudo mixed-cell expression data is generated taking n_p random samples (with replacement) of 2 to 8 cells from the scRNA-seq reference, and aggregating their UMI counts, downsampling to adjust for realistic bulk UMI counts. The pseudo and real mixed-cell data are then aligned in a lower dimensional ($S < d$) gene-space using Canonical Correlation Analysis (CCA). The projections to the $s = 1, 2, ..., S$ dimensions are given by the canonical variables

$$\begin{aligned} U_s &= \widetilde{X}\mu_s^* \\ V_s &= X\nu_s^* \end{aligned} \tag{71}$$

where

$$\mu_s^*, \nu_s^* = \underset{\mu_s, \nu_s \in \mathbb{R}^d}{argmax}\{\nu_s^T \widetilde{X}^T X \nu_s\} \text{ s.t. } U_s^T U_{s'} = V_s^T V_{s'} = \delta_{ss'} \tag{72}$$

are the canonical correlation vector pairs. These embeddings are then used to construct a graph by considering Mutual Nearest Neighbors (MNN) as adjacent in the graph. That is, given a pair of sample cell-pools i, j, we let

$$A_{i,j} = \begin{cases} 1 & \text{if } i \text{ and } j \text{ are mutual nearest neighbors} \\ 0 & \text{otherwise} \end{cases} \tag{73}$$

Here, adjacencies can be between simulated-to-real and real-to-real samples. With $X_{in} = [\widetilde{X} X] \in \mathbb{R}^{d\times N}$ ($N = n_p + n$) and the normalized adjacency matrix $\widetilde{A}$ as input, the $L \geq 1$ (default 1) graph convolution (GCN) layers of the DSTG model are given by

$$\begin{aligned} H^{(0)} &= X_{in} \\ H^{(l)} &= \text{ReLU}(\widetilde{A}H^{(l-1)}W^{(l)}) \text{ for } l \in [1, L] \end{aligned} \tag{74}$$

where $W^{(l)}$ is the weight matrix for the l_{th} layer. The output of the DSTG model is the predicted composition of K cell-types, given by

$$\begin{bmatrix} \hat{Y}_p \\ \hat{Y} \end{bmatrix} = \text{softmax}(\widetilde{A}H^{(L)}W) \in \mathbb{R}^{N\times K} \tag{75}$$

where $\hat{Y}_p$ and $\hat{Y}$ are the predictions for the pseudo and real cell-pools, respectively. The loss function is then defined as the cross-entropy between the predicted and true cell-type compositions of the pseudo cell-pools

$$\mathcal{L} = -\sum_{i=1}^{n_p}\sum_{k=1}^{K} y_{i,k}^{(p)} ln(\hat{y}_{i,k}^{(p)}) \tag{76}$$

where $\hat{y}_{i,k}^{(p)}$ and $y_{i,k}^{(p)}$ are the predicted and true composition of cell-type k in the i_{th} pseudo cell-pool.

dance.modules.spatial.cell_type_deconvo.spotlight SPOTlight [76] builds on the classic non-negative least squares approach to cell-type deconvolution by incorporating topic-modeling for both the reference scRNA-seq expression data $X_s \in \mathbb{R}^{d\times N}$ and the mixed-cell expression data $X \in \mathbb{R}^{d\times n}$. First, non-negative matrix factorization (NMF) is applied to the reference X_s to get

$$W, H = \underset{W', H' \geq 0}{argmin} \left\| X_s - W'H' \right\|_F \tag{77}$$

where the rows of $H \in \mathbb{R}^{K \times N}$ are the cell-topic embeddings, and the columns of $W \in \mathbb{R}^{d \times K}$ the corresponding weightings. Cell-topic profiles $\widetilde{H} \in \mathbb{R}^{K \times K}$ are then constructed from H by taking the median over each cell-type. Next, spot-topic profiles $P \in \mathbb{R}^{K \times n}$ are constructed through NNLS of X onto W

$$P = \underset{P' \geq 0}{argmin} \|X - WP'\|_F \tag{78}$$

Finally, the estimator of cell-type compositions for the n cell-pools is then given by

$$\widehat{Y} = \underset{B \geq 0}{argmin} \|P - \widetilde{H}B\|_F \tag{79}$$

dance.modules.spatial.cell_type_deconvo.spatialdecon SpatialDecon [77] is non-negative linear regression based method that assumes a log-normal multiplicative error model between the mixed-cell data $X \in \mathbb{R}^{d \times n}$ and a cell-profile (signature) matrix $\widetilde{X} \in \mathbb{R}^{d \times K}$. The cell-profile matrix $\widetilde{X}$ is a measure of center (median by default) expression for each of the K cell-types, constructed from the reference scRNA-seq data $X_s \in \mathbb{R}^{d \times N}$. The log-normal multiplicative error model is given by

$$log(X_{i\cdot}) = log(\widetilde{X}_{i\cdot}^T B) + \epsilon_i, \text{ where } \epsilon_i \sim \mathcal{N}(0, \sigma^2 I_n) \text{ and } B \in \mathbb{R}^{K \times n} \tag{80}$$

The estimator of cell-type compositions for the n cell-pools is then given by

$$\hat{Y} = \underset{B \geq 0}{argmin} \left\| log(X) - log(\widetilde{X}^T B) \right\|_2 \tag{81}$$

dance.modules.spatial.cell_type_deconvo.card CARD [78] applies a conditional autoregressive (CAR) assumption on the coefficients of the classical non-negative linear model between the mixed-cell expression X and a cell-profile matrix $\widetilde{X}_s$, constructed from reference scRNA-seq X_s. The linear model is given by

$$X = \widetilde{X}_s B + \epsilon, \epsilon \sim \mathcal{N}(0, \sigma_e^2 I_n) \tag{82}$$

The CAR assumption then incorporates 2D spatial information $S = \begin{bmatrix} s_1 \\ \vdots \\ s_n \end{bmatrix} \in \mathbb{R}^{n \times 2}$ through an intrinsic prior on the cell-type compositions (the model coefficients) by modeling compositions in each location as a weighted combination of compositions in all other locations. This modeling assumption is given by

$$B_{ki} = b_k + \phi \sum_{j=1, j \neq i}^{n} W_{ij}(B_{kj} - b_k) + \epsilon_{ki}, \epsilon_{ki} \sim \mathcal{N}(0, \sigma_{ki}^2) \tag{83}$$

where the weights W_{ij} are given by the Gaussian kernel

$$W_{ij} = K_G(s_i, s_j; \sigma^2) = \exp(-\frac{\|s_i - s_j\|_2^2}{2\sigma^2}) \tag{84}$$

with default scaling parameter $\sigma^2 = 0.1$. CARD then estimates the cell-type composition of the n cell-pools through constrained maximum likelihood estimation.
