# Supplementary material for: DANCE: a deep learning library and benchmark platform for single-cell analysis
Source: Genome Biol. 2024 Mar 19;25:72. doi: 10.1186/s13059-024-03211-z (PMC10949782; doi:10.1186/s13059-024-03211-z)
Supplement: Supplementary file 2 — Additional file 2. Appendix B — Environment Dependencies in DANCE. [file 13059_2024_3211_MOESM2_ESM.pdf]

Appendix B: Environment Dependencies in DANCE

For the dependencies of our first version of the DANCE package, please refer to Table 3 for more details.

Dependency	Version
h5py	$\geq$3.7.0
leidenalg	$\geq$0.8.10
networkx	$\geq$2.8.5
numba	$\geq$0.56.0
opencv-python	$\geq$4.6.0.66
openpyxl	$\geq$3.0.10
psutil	$\geq$5.9.1
pyro-ppl	$\geq$1.8.1
python-igraph	$\geq$0.9.11
rdata	$\geq$0.8
scanpy	$\geq$1.9.1
scikit-learn	$\geq$1.1.2
scikit-misc	$\geq$0.1.4
scipy	$\geq$1.9.0
seaborn	$\geq$0.11.2
skorch	$\geq$0.11.0
statsmodels	$\geq$0.13.2
torch	$\geq$1.11.0
torchnmf	$\geq$0.3.4
torchvision	$\geq$0.12.0
tqdm	$\geq$4.64.0

Table 3: Dependencies of DANCE Package
