# Supplementary material for: DANCE: a deep learning library and benchmark platform for single-cell analysis
Source: Genome Biol. 2024 Mar 19;25:72. doi: 10.1186/s13059-024-03211-z (PMC10949782; doi:10.1186/s13059-024-03211-z)
Supplement: Supplementary file 3 — Additional file 3. Appendix C — Codebase Structure Tree in DANCE. [file 13059_2024_3211_MOESM3_ESM.pdf]

Appendix C: Codebase Structure Tree in DANCE

The structure tree below shows our codebase structure in DANCE. Basically it consists of two parts: dance source code and examples. In example folder, each file represent one example to show how to leverage one specific model on dataset.

```
|___    LICENSE
|___    README.md
|___    dance
|   |___    __init__.py
|   |___    data
|   |   |___    __init__.py
|   |___    datasets
|   |   |___    __init__.py
|   |   |___    multimodality.py
|   |   |___    singlemodality.py
|   |   |___    spatial.py
|   |___    modules
|   |   |___    __init__.py
|   |   |___    multi_modality
|   |   |   |___    __init__.py
|   |   |   |___    joint_embedding
|   |   |   |   |___    __init__.py
|   |   |   |   |___    dcca.py
|   |   |   |   |___    jae.py
|   |   |   |   |___    scmogcn.py
|   |   |   |   |___    scmogcnv2.py
|   |   |   |   |___    scmvae.py
|   |   |   |___    match_modality
|   |   |   |   |___    __init__.py
|   |   |   |   |___    cmae.py
|   |   |   |   |___    scmm.py
|   |   |   |   |___    scmogcn.py
|   |   |   |___    predict_modality
|   |   |       |___    __init__.py
|   |   |       |___    babel.py
|   |   |       |___    cmae.py
|   |   |       |___    scmm.py
|   |   |       |___    scmogcn.py
|   |   |___    single_modality
|   |   |   |___    __init__.py
|   |   |   |___    cell_type_annotation
|   |   |   |   |___    __init__.py
|   |   |   |   |___    actinn.py
|   |   |   |   |___    celltypist.py
|   |   |   |   |___    scdeepsort.py
|   |   |   |   |___    singlecellnet.py
|   |   |   |   |___    svm.py
|   |   |   |___    clustering
|   |   |   |   |___    __init__.py
|   |   |   |   |___    graphsc.py
|   |   |   |   |___    scdcc.py
|   |   |   |   |___    scdeepcluster.py
|   |   |   |   |___    scdsc.py
|   |   |   |   |___    sctag.py
|   |   |   |___    imputation
|   |   |       |___    __init__.py
|   |   |       |___    deepimpute.py
|   |   |       |___    graphsci.py
|   |   |       |___    scgnn.py
|   |   |___    spatial
|   |       |___    __init__.py
|   |       |___    cell_type_deconvo
|   |       |   |___    __init__.py
|   |       |   |___    card.py
|   |       |   |___    dstg.py
|   |       |   |___    spatialdecon.py
|   |       |   |___    spotlight.py
|   |       |___    spatial_domain
|   |           |___    __init__.py
|   |           |___    louvain.py
|   |           |___    spagcn.py
```

```
|   |           |___    stagate.py
|   |           |___   stlearn.py
|   |___    plotting
|   |   |___   __init__.py
|   |___    transforms
|   |   |___    __init__.py
|   |   |___    graph_construct.py
|   |   |___   preprocess.py
|   |___   utils
|       |___    __init__.py
|       |___    loss.py
|       |___   metrics.py
|___    docs
|   |___    Makefile
|   |___    make.bat
|   |___    requirements.txt
|   |___   source
|       |___    conf.py
|       |___    index.rst
|       |___   modules
|           |___    multi_modality.rst
|           |___    single_modality.rst
|           |___   spatial.rst
|___    examples
|   |___    multi_modality
|   |   |___    joint_embedding
|   |   |   |___    dcca.py
|   |   |   |___    jae.py
|   |   |   |___    scmogcn.py
|   |   |   |___    scmogcnv2.py
|   |   |   |___   scmvae.py
|   |   |___    match_modality
|   |   |   |___    cmae.py
|   |   |   |___    scmm.py
|   |   |   |___   scmogcn.py
|   |   |___   predict_modality
|   |       |___    babel.py
|   |       |___    cmae.py
|   |       |___    scmm.py
|   |       |___   scmogcn.py
|   |___    single_modality
|   |   |___    cell_type_annotation
|   |   |   |___    actinn.py
|   |   |   |___    celltypist.py
|   |   |   |___    scdeepsort.py
|   |   |   |___    singlecellnet.py
|   |   |   |___   svm.py
|   |   |___    clustering
|   |   |   |___    graphsc.py
|   |   |   |___    scdcc.py
|   |   |   |___    scdeepcluster.py
|   |   |   |___    scdsc.py
|   |   |   |___   sctag.py
|   |   |___   imputation
|   |       |___    deepimpute.py
|   |       |___    graphsci.py
|   |       |___   scgnn.py
|   |___   spatial
|       |___    cell_type_deconvo
|       |   |___    card.py
|       |   |___    dstg.py
|       |   |___    spatialdecon.py
|       |   |___   spotlight.py
|       |___   spatial_domain
|           |___    louvain.py
|           |___    spagcn.py
|           |___    stagate.py
|           |___   stlearn.py
|___    imgs
|   |___    dance_logo.jpg
|   |___   framework.png
|___    install.sh
```

```
|___     pyproject.toml
|___     requirements.txt
|___     setup.cfg
|___     setup.py
|___     tests
|   |___    test_bench.py
|___    tox.ini
```
