# Supplementary material for: DANCE: a deep learning library and benchmark platform for single-cell analysis
Source: Genome Biol. 2024 Mar 19;25:72. doi: 10.1186/s13059-024-03211-z (PMC10949782; doi:10.1186/s13059-024-03211-z)
Supplement: Supplementary file 4 — Additional file 4. Appendix D — Contribution Instructions in DANCE. [file 13059_2024_3211_MOESM4_ESM.pdf]

Appendix D: Contribution Instructions in DANCE

DANCE is an open-source package, and everyone can contribute to this platform with extra tasks, models, and standard benchmark datasets by following the instructions below:

- **Codebase Structure Guidance:** Before contributing to DANCE, you need to understand the codebase structure in DANCE and make sure you are going to modify the correct files or put new files into the correct place. dance is the root of the package. datasets is the dataloader module, which contains task specific data loaders. modules is the model module, which covers all implemented algorithms. Each model is created as an individual file under the corresponding task folder. transforms is the data processing module, which deals with data preprocessing and graph construction. You are flexible to pick up any existing functionalities under this folder for your model development and welcome to contribute new ones if your desired ones are not provided. examples is the example reference module, which presents one example for one model.
- **Code Style Guidance:** The contributed codes are required to follow the standard python coding style. For more details, please refer to Style Guide for Python Code.
- **Testing:** To ensure that your contributed code does not impact the normal operation of the existing codebase, you need to ensure that all tests pass before submitting. Please refer to the **Run Test** section in DANCE for how to run tests.
- **Reproducibility:** This is only required for contributed new models. An example file is necessary to present how to run your model on existing standard benchmarks. Furthermore, you need to place command lines at the end of the example file to obtain the best performance for reproducibility purposes. One command line corresponds to one standard benchmark dataset running of your contributed model.
- **Documentation:** Submitted code should be documented or commented on for easy readability purposes by users. Please refer to Numpy Style Docstring Guide for more details.
