# Supplementary material for: DANCE: a deep learning library and benchmark platform for single-cell analysis
Source: Genome Biol. 2024 Mar 19;25:72. doi: 10.1186/s13059-024-03211-z (PMC10949782; doi:10.1186/s13059-024-03211-z)
Supplement: Supplementary file 5 — Additional file 5. Appendix E — More Performance Showup in DANCE. [file 13059_2024_3211_MOESM5_ESM.pdf]

Appendix E: More Performance Showup in DANCE.

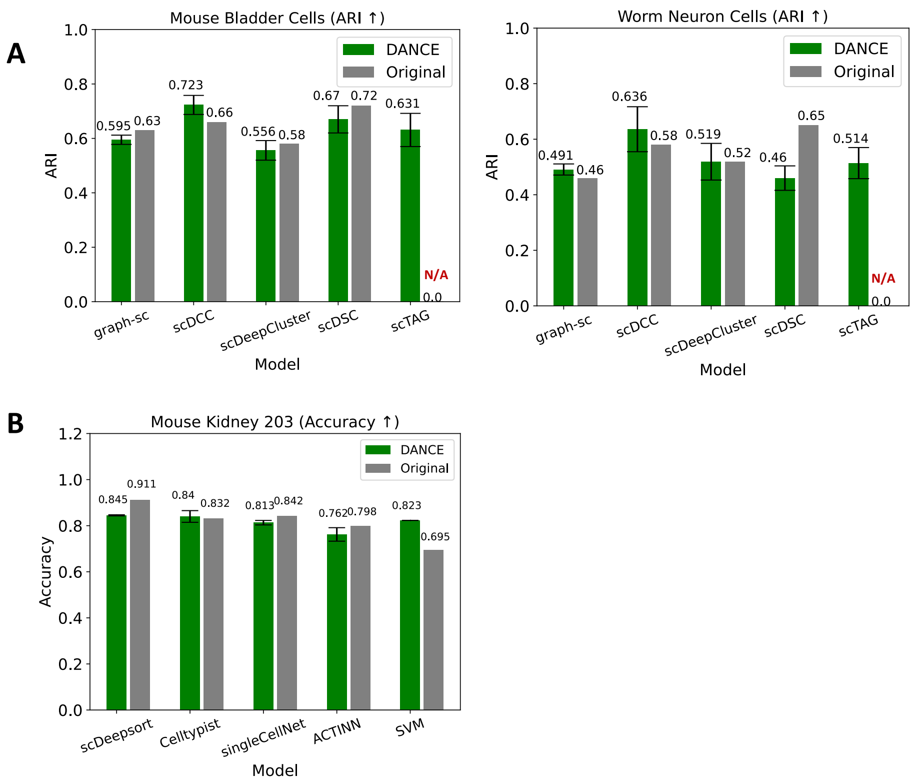

Figure 9: The performance comparison between our implementation and original implementation for supported tasks in the single-modality module. DANCE result represents the mean performance across 20 randomly chosen seeds, while the Original result refers to the performance directly extracted from the original paper. **a** Clustering task. **b** Cell type annotation task. NOTE: N/A indicates no performance report from the original paper.

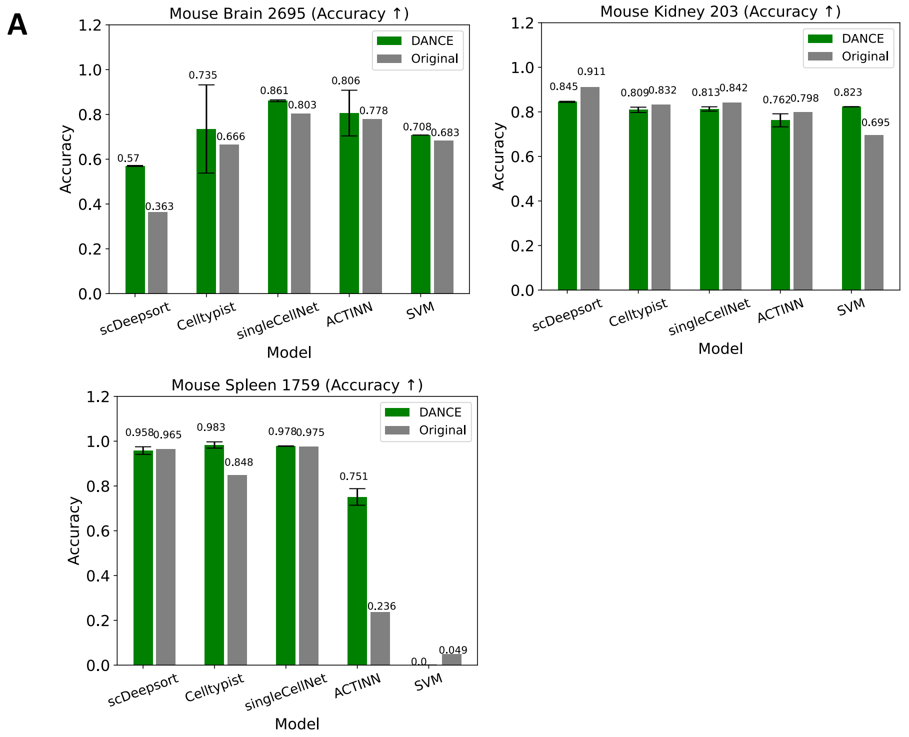

Figure 10: The performance comparison between our implementation and original implementation for cell type annotation (majority_voting as True in Celltypist). DANCE result represents the mean performance across 20 randomly chosen seeds, while the Original result refers to the performance directly extracted from the original paper. **a** Cell Type Annotation task. NOTE: N/A indicates no performance report from the original paper.

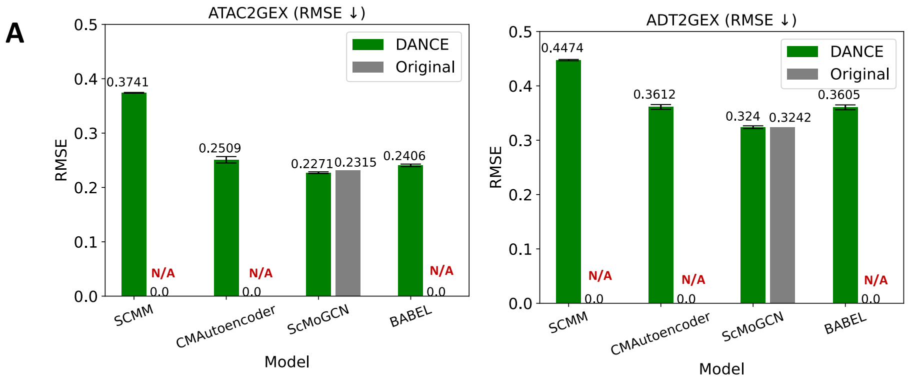

Figure 11: The performance comparison between our implementation and original implementation for supported tasks in the multi-modality module. DANCE result represents the mean performance across 20 randomly chosen seeds, while the Original result refers to the performance directly extracted from the original paper. **a** Modality prediction task. NOTE: N/A indicates no performance report from the original paper.

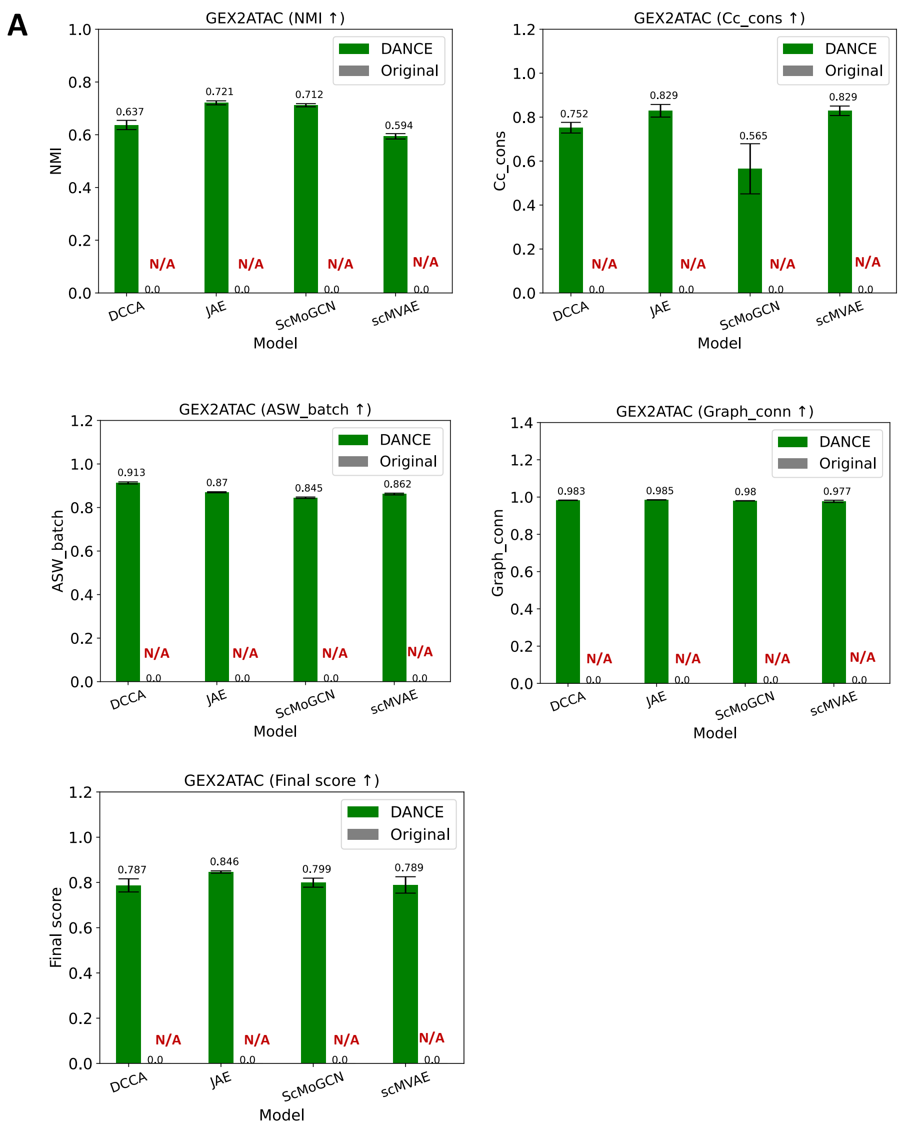

Figure 12: The performance comparison between our implementation and original implementation for supported tasks in the multi-modality module. DANCE result represents the mean performance across 20 randomly chosen seeds. **a** more metrics evaluated in the Joint Embedding task. The final score indicates the average score among all metrics. NOTE: N/A indicates no performance report from the original paper.

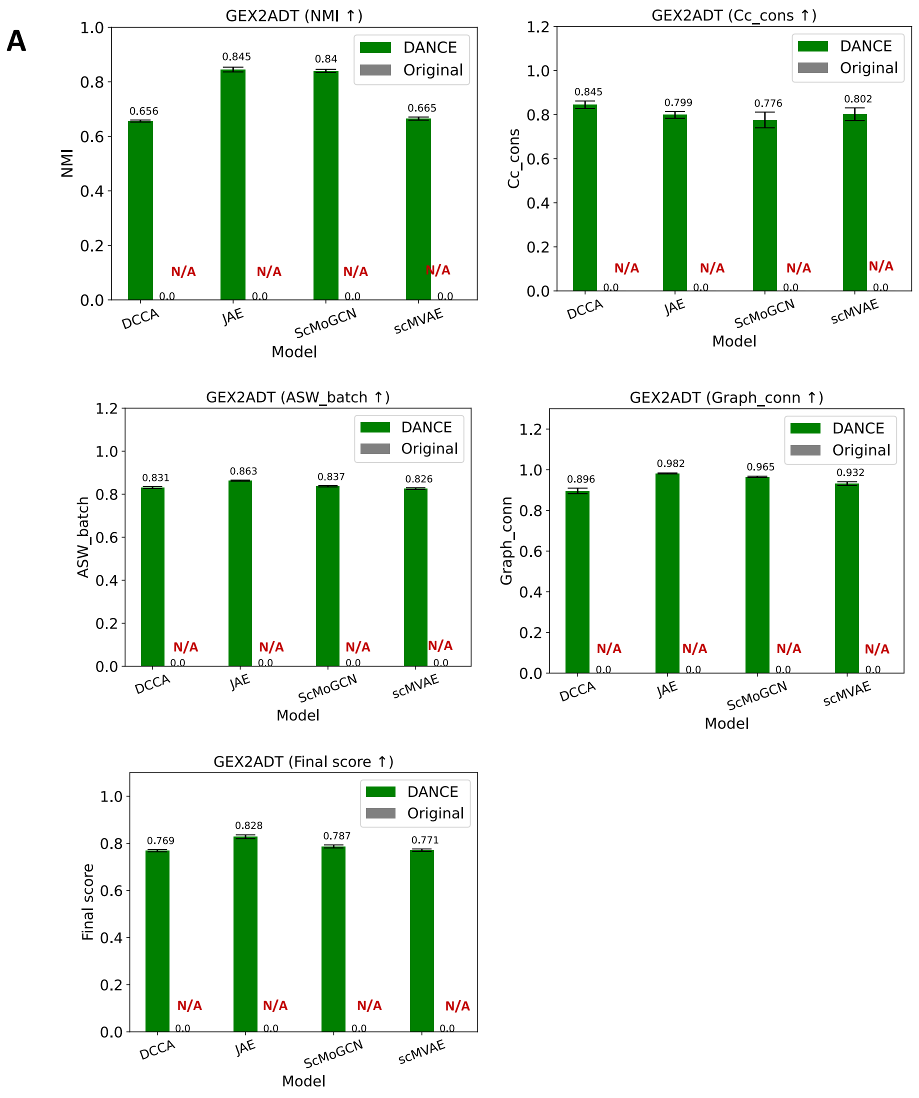

Figure 13: The performance comparison between our implementation and original implementation for supported tasks in the multi-modality module. DANCE result represents the mean performance across 20 randomly chosen seeds. **a** more metrics evaluated in the Joint Embedding task. The final score indicates the average score among all metrics. NOTE: N/A indicates no performance report from the original paper.

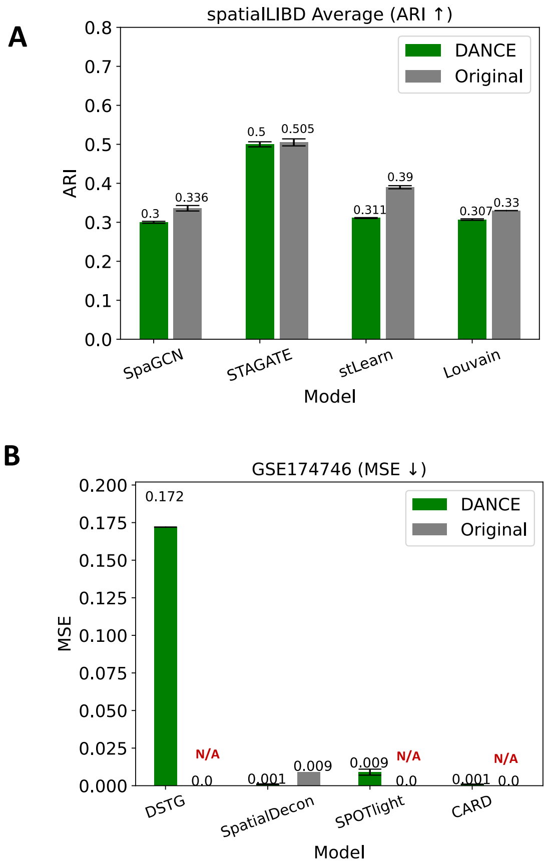

Figure 14: The performance comparison between our implementation and original implementation for supported tasks in the spatial transcriptomics module. DANCE result represents the mean performance across 20 randomly chosen seeds, while the Original result refers to the performance directly extracted from the original paper. **a** Spatial domain identification task. Average performance on 12 slices on spatialLIBD dataset. **b** Cell type deconvolution task. NOTE: N/A indicates no performance report from the original paper.
