# Supplementary material for: DANCE: a deep learning library and benchmark platform for single-cell analysis
Source: Genome Biol. 2024 Mar 19;25:72. doi: 10.1186/s13059-024-03211-z (PMC10949782; doi:10.1186/s13059-024-03211-z)
Supplement: Supplementary file 6 — Additional file 6. Review history. [file 13059_2024_3211_MOESM6_ESM.docx]

**1^st^ round**

**Reviewer 1**

The authors offer a single-cell deep learning library (DANCE) for the community of single cell biology researchers. They claim that the library can help benchmark some tasks and the results are reproducible with optimized hyper-parameters. Besides, they also claim that DANCE can help facilitate the algorithms development by providing various state-of-the-art deep learning tools.

Major comments:

1. Data format issue. In the python single cell tools community, popular tools like SCANPY, scVI-tools, scVelo… will provide data in AnnData (https://anndata.readthedocs.io/en/latest/) which is more convenient compared to .csv tables. I suggest DANCE should also prepare the benchmarking datasets in this format (or muon.mudata format for multiomics datasets) and provide API for researchers to download. Please refer to https://scanpy.readthedocs.io/en/stable/api.html#module-scanpy.datasets.

2. Detailed tutorials and API notes are necessary. The library docs only provide partial API annotation right now (March 31, 2023), it is not user-friendly if users can only find annotations in source code. For the dataset loader part, it is also confusing. I suggest DANCE should provide a detailed tutorial to guide users constructs dataset and data loader from their own AnnData.

3. This library is not a reproducible benchmarking platform. For some methods, you forget to set random seed in the example script. And, more importantly, you should pin the random seed and report the performance error bar with at least 20 random seeds. By the way, the DGL library now has problem in dgl.seed() function, it is not compatible with the newest PyTorch, you may test it and warn it in the installation guide.

4. In Figure 3, why some methods' original performance is NA and some methods' are not? I don't think original SVM paper has a cell type classification accuracy report. You should try your best to run these methods to show the differences rather than reporting NA. If you don't report the performance differences between your implementation and original one's, how can I believe your implementation is right? Also, code used to test the original method is required to post.

5. Evaluation Metrics. For the NeurIPS datasets, one important task is to integrate different batches. SO, the evaluation metrics should include some conventional metrics used to evaluate batch correction. The metrics has been implemented by scIB (https://scib.readthedocs.io/en/latest/). Page 19, Line 45, as far as I know, the competition requires the latent dim <= 100 instead of = 100.

6. Scalability. One concern is that, how about the new implementation's scalability compared to the original one's?

7. I am afraid that the authors do not fully understand the value of original methods? Since I am familiar with celltypist, I tested the implementation of yours but find two major problems. First, celltypist highly relies on the majority voting process but you set the action="store_true", I hope you know that, store_true means False in argparser. Second, celltypist utilize the pretrained mode to make prediction on new data. But the users can not access a pretrained model from DANCE.


8. Is it really possible to facilitate algorithms building process? DANCE does not provide useful autoencoder interface and customized options.


Minor Comments:

1. Table 1, what is the meaning of Benchmark datasets and task specific algorithms? At least scanpy and seurat contain some datasets. Is Leiden / Louvain a task specific algorithms? If so, scanpy & seurat should also have a mark.

2. Why the dataset class for cell type annotation task is named as scDeepSort datasets? It's weird.

**Reviewer 2**

In this work, the authors present DANCE, a standardized, comprehensive, and extensible benchmarking platform that can be used to evaluate various benchmark datasets and single-cell analysis tasks. The authors evaluated 3 modules, 8 tasks, 32 models, and 21 benchmark datasets and people can easily reproduce the results of supported algorithms across major benchmark datasets only with one command line. DANCE implements all models in a unified development environment based on the Python language, using Pytorch, the Depth Graph Library (DGL), and PyTorch Geometric (PyG) as the backbone framework. Based on a number of evaluation metrics, the authors conclude that the performance of the newly implemented models based on DANCE is equivalent or even superior to that of the models in the original papers. Overall, this paper provides a benchmark platform to facilitate research and development in single-cell analysis. The authors employ suitable methods and pose a clear research question. However, there are some concerns that should be addressed.

Major Comments:
1. The manuscript briefly mentions that DANCE provides a variety of ways for graph construction. The weight of each edge between each cell node and its corresponding gene node is determined by gene counts, and there is no edge linked between any pairs of cell or gene nodes. However, the details of constructing the weighted heterogeneous cell-to-gene graph are not clearly explained. It will be better if they could provide more explanations for the construction of graphs.

2. In the Single modality module - Cell type annotation on page 11, the authors mention that the performance of their SingleCellnet and ACTINN is lower than the reported performance from the paper on Mouse Brain 2695. And the accuracy of SingleCellnet and ACTINN is also lower in the additional file 5 Figure 1: b. What is the cause of this state of things? The authors should also consider trying more datasets or multiple metrics to evaluate their methods.

3. In the manuscript on page 14, lines 42-44. The authors state that most of the models' performances on two slices are not reported individually in the original paper. This statement is inaccurate, the performance on 12 slices is compared inside these model supplement materials. The authors only provide a comparison of performance on the 151507, 151673, and 151676 slices, and there is no improvement in the ARI of 151673 in Additional file 5 Figure 3: a. The authors should consider more convincing performance comparisons in all 12 slices of the DLPFC dataset.

4. Table 2 summarizes all supported benchmark datasets in DANCE. What are the criteria for benchmarking data selection? As sequencing technology advances and more single-cell datasets become available, how can this model be applied to new datasets？

5. Could DANCE support the addition of R language-based models for benchmarking? And if so, the applicability of the models will be improved. How to perform hyperparameter tuning experiments on the newly added model?

6. It will be better if the authors could make their experiment data open access.

Minor comments:
1. Please ensure that all abbreviations are fully defined and explained. Where an English term abbreviation first appears in the manuscript, the corresponding full name should be written first. For example, Page 12 line 52 "GEX".

2. When an abbreviation is defined and then reappears in the manuscript, use the abbreviation, not the full name. For example, "Deep Graph Library (DGL) and PyTorch Geometric (PyG)" appear several times in the manuscript.

3.It is suggested that future directions and potential areas of improvement be provided in the conclusion section to consider additional single-cell analysis tasks to further enhance the significance and practical utility of the DANCE.

**Authors’ response**

**Response to both Reviewer #1 and Reviewer #2**

For review purposes, please refer to the **https://github.com/OmicsML/dance/tree/v1.0.0-benchmark** branch of the repository, where we conducted the 20 random seeds experiments as suggested by the  reviewer. We are working on some minor incompatibilities between this branch and the main branch  and will merge over these changes once done.

During the last review period, our DANCE team also hosted a HANDS-ON LAB, LIVE TUTORIAL on June  29th via Zoom to guide users how to use DANCE including DANCE environment set up, data loading and  processing, basic deep learning framework walk through, example methods providing a detailed, step by-step tutorial for each task. The LIVE TUTORIAL attracted a lot of participation. Below we provide the  links to the HANDS-ON LAB and the recording to the event.

● DANCE online tutorial Github repository: https://github.com/OmicsML/dance-tutorials ● DANCE online tutorial YouTube recording: https://www.youtube.com/watch?v=xXzmx4KIvYo

**Reviewer #1**

**Reviewer’s Comment**: Major 1. Data format issue. In the python single cell tools community, popular  tools like SCANPY, scVI-tools, scVelo… will provide data in AnnData

(https://anndata.readthedocs.io/en/latest/) which is more convenient compared to .csv tables. I suggest  DANCE should also prepare the benchmarking datasets in this format (or muon.mudata format for  multiomics datasets) and provide API for researchers to download. Please refer to  https://scanpy.readthedocs.io/en/stable/api.html#module-scanpy.datasets.

**Authors’ Response:** We thank the reviewer for the valuable suggestion. We have therefore  improved our main DANCE data object to interface with the AnnData object seamlessly. Specifically,  now the DANCE data object directly takes AnnData (or MuData for multi-modality data) as the main  input for construction. We have additionally provided a short tutorial for the DANCE data object,  including how to use the built-in datasets, as well as how to construct a DANCE dataset object using  user-given AnnData (https://github.com/OmicsML/dance/blob/v1.0.0-

benchmark/notebooks/dance_data_tutorial.ipynb).

**Reviewer’s Comment**: Major 2. Detailed tutorials and API notes are necessary. The library docs only  provide partial API annotation right now (March 31, 2023), it is not user-friendly if users can only find  annotations in source code. For the dataset loader part, it is also confusing. I suggest DANCE should  provide a detailed tutorial to guide users constructs dataset and data loader from their own AnnData.

**Authors’ Response:** We appreciate the reviewer’s suggestion regarding the package’s API  documentation and tutorial. As per the v1.0.0 release of the DANCE package, we have significantly  improved the documentation hosted on ReadTheDocs (https://pydance.readthedocs.io/en/latest/),  which covers all primary tasks (single modality, multi-modality, spatial) and modules (data, datasets,  transforms). In addition, we have provided a detailed tutorial notebook that can be launched from  Google Colab in a dedicated repository (https://github.com/OmicsML/dance-tutorials/blob/tutorial v1/dance_tutorial.ipynb). In the future, we plan to continually update this tutorial repository as more  features and models are being added.

**Reviewer’s Comment**: Major 3. This library is not a reproducible benchmarking platform. For some  methods, you forget to set random seed in the example script. And, more importantly, you should pin  the random seed and report the performance error bar with at least 20 random seeds. By the way, the  DGL library now has problem in dgl.seed() function, it is not compatible with the newest PyTorch, you  may test it and warn it in the installation guide.

**Authors’ Response:** We appreciate the reviewer's valuable feedback. We have carefully reviewed  and improved our benchmarking scripts, ensuring random seeds are set consistently using our  "set_seed" utility function, which covers both torch and DGL seeds. Moreover, we have rerun **all** experiments reported from DANCE with 20 random seeds and have reported performance with error  bars in the revised manuscript.

Regarding the DGL library's compatibility with the latest PyTorch, we have not encountered any issues  while using DGL version 1.1.2 with PyTorch version 2.0.1. However, to address the reviewer's concerns,  we kindly request more specific details or links to discussions or issues related to DGL's random seed  compatibility issue with PyTorch. This information would help us further investigate the matter to  adequately address the potential problem raised in the reviewer's original comment.

**Reviewer’s Comment**: Major 4. In Figure 3, why some methods' original performance is NA and some  methods' are not? I don't think original SVM paper has a cell type classification accuracy report. You  should try your best to run these methods to show the differences rather than reporting NA. If you don't  report the performance differences between your implementation and original one's, how can I believe  your implementation is right? Also, code used to test the original method is required to post.

**Authors’ Response:** We thank the reviewer sincerely for reviewing our study. NA means the original  paper doesn’t report performance on this dataset. Yes, you are right. In the original SVM paper, there is  no performance report for cell type annotation. In one popular benchmark paper (“A comparison of  automatic cell identification methods for single-cell RNA sequencing data”), it reports the cell type  annotation performance from SVM, where we got the reported performance from SVM.

For performance of original implementation, we just directly picked up the reported performance from  the paper. The performance reported in the original paper can be compared with our implementation  on the same dataset to improve our implementation’s efficacy.

From most results shown in the manuscript, our performance is similar to the original and sometimes  even better. But sometimes ours is worse, and the differences may arise from language implementation  (python VS R), python version, package version, operating systems, and other reasons.

We are trying our best to make the difference as small as possible and make the performance of our  reimplementation more convincing via collecting more benchmark datasets for comprehensive  evaluation and executing more metric evaluation as we did for multimodal integration in the revision.  Taking joint embedding tasks as an example, we add additional Biology Conservation Metrics and Batch  Removal Metrics for comprehensive evaluation in the revised manuscript.

Biology Conservation Metrics:

● Normalized mutual information (NMI) metric: compares the overlap of two clusterings ● Cell cycle conservation score metric: serves as a proxy for the preservation of the signal  associated with gene programs during data integration.

Batch Removal Metrics:

● Batch ASW metric: is used to quantify batch mixing by taking into account the incompatibility of  batch labels per cell type cluster.

● Graph connectivity metric: determines whether cells of the same kind from various batches are  embedded close to one another.

Please refer to the joint embedding task in the revised manuscript for more performance comparisons  under different metrics.

**Reviewer’s Comment**: Major 5. Evaluation Metrics. For the NeurIPS datasets, one important task is  to integrate different batches. SO, the evaluation metrics should include some conventional metrics  used to evaluate batch correction. The metrics has been implemented by scIB

(https://scib.readthedocs.io/en/latest/). Page 19, Line 45, as far as I know, the competition requires the  latent dim <= 100 instead of = 100.

**Authors’ Response:** We appreciate the reviewer's valuable feedback. Our DANCE team actually  attended the 2021 NeurIPS competition and got first place in the task of Modality Prediction. The  winning solution proposed by our DANCE team is called ScMoGCN, which is currently supported by  DANCE. In the revised manuscript, we have additionally included Biology Conservation Metrics and  Batch Removal Metrics for comprehensive evaluation under Joint Embedding task as mentioned in the  last review response.

For the joint embedding task in the competition, the latent dimension is set to be less than or equal to  100. Based on our experiments, we found that the latent dimension setting to be 100 always achieves  better performance since higher dimension carries more information. That’s the reason we set it to be  100. We have corrected the statement (latent dim <= 100) to be consistent with the description from

the NeurIPS competition in the revised manuscript. Thank you for pointing this out.

**Reviewer’s Comment**: Major 6. Scalability. One concern is that, how about the new  implementation's scalability compared to the original one's?

**Authors’ Response:** We really appreciate the reviewer for pointing out this insightful question. The  scalability is one main motivation when we designed the DANCE platform. With the rapid growth of  single-cell data, we think that the single-cell methods based on R language cannot quickly process  millions or more data as we expect, so we established DANCE, a unified development environment  based on python language with Pytorch. Python and deep learning libraries exhibit notable advantages  in facilitating GPU acceleration, particularly when dealing with a substantial quantity of single-cell data.

As we can see from the figure above, under the acceleration of GPU, our implementation (SpaGCN  (GPU) and STATGATE (GPU)) in DANCE exhibits greater advantage compared to CPU running as the  quantity of single-cell data increases. Some of the original implementation does not support GPU  acceleration but we support all of them under GPU running if only they are Neural Network based  methods.

**Reviewer’s Comment**: Major 7. I am afraid that the authors do not fully understand the value of  original methods? Since I am familiar with celltypist, I tested the implementation of yours but find two  major problems. First, celltypist highly relies on the majority voting process but you set the  action="store_true", I hope you know that, store_true means False in argparser. Second, celltypist utilize  the pretrained mode to make prediction on new data. But the users can not access a pretrained model  from DANCE.

**Authors’ Response:** We appreciate the reviewer’s comment regarding the usage of CellTypist. First,  we follow the CellTypist API strictly, which turns off the majority_voting option by default (see  https://github.com/Teichlab/celltypist/blob/0a5e319278d4d4e64ea8d3c57596a24d47071075/celltypist /annotate.py#L16). Thus, we are aware of our intended setting of majority_voting being False by default  through the command line interface. We do so based on the original API’s preference. However, we  have also included the cell type annotation benchmarking results for CellTypist, with majority voting  turned on, to accommodate the reviewer’s suggestion. We found that the majority voting could improve  the performance, but this is dataset-dependent. We have included cell type annotation benchmarking  results for CellTypist, with majority voting turned on in the revised manuscript (Additional file 5 Figure  2). Thank the reviewer again for careful reading and for your patience, which we greatly appreciate.

Second, we thank the reviewer for suggesting using pre-trained models, using CellTypist as an example.  This is a really insightful suggestion. We are currently working on preparing comprehensive support for  pre-trained models, from simple models like CellTypist to more advanced transformers like scGPT. We  are very excited about this upcoming feature, but we note that this is ultimately out of the scope of the  current version of DANCE. We thank the reviewer for understanding this, and please keep attention on  this upcoming feature we will be putting out soon.

**Reviewer’s Comment**: Major 8. Is it really possible to facilitate algorithms building process? DANCE  does not provide useful autoencoder interface and customized options.

**Authors’ Response:** We thank the reviewer for the question and the opportunity for us to clarify  this matter. We believe that DANCE is indeed a useful resource that facilitates algorithm building, but  not merely in the sense we have alluded initially to, which primarily argued about providing readily plug and-playable components for new models. Designing modularized components that are fully compatible  across diverse tasks is not straightforward, and we admit that we have not completely and satisfactorily  delivered this exact feature. However, we highlight DANCE's value in facilitating new single-cell  algorithm building, but for different reasons.

1. **Standardized Framework**: DANCE provides a standardized framework for implementing any  new methods in diverse types of tasks, including single/multi-modality and spatial analysis. Our  BaseMethod class provides a unified and object oriented structure for implementing new  methods for the above tasks

(https://github.com/OmicsML/dance/blob/212a055a957c9238242ecb238b8311f8e9fbe4c6/dan ce/modules/base.py#L17). Following our blueprint, researchers can focus on the model  development part by only implementing the fit and predict functions, while all other non-model  related handling is taken care of by the framework. This simplifies and accelerates algorithm  development.

2. **Advanced Preprocessing**: DANCE provides extensive preprocessing functions, surpassing  standard options like library size normalization and log transformation available in scanpy. These  include advanced and highly specialized transformations such as (i) the differential gene-pair feature designed and used by SingleCellNet (https://github.com/OmicsML/dance/blob/v1.0.0- benchmark/dance/transforms/scn_feature.py), (ii) heterogeneous cell-gene graph construction  used by scDeepSort (https://github.com/OmicsML/dance/blob/v1.0.0-

benchmark/dance/transforms/graph/cell_feature_graph.py), (iii) cell morphology features  derived from histology images used by stLearn

(https://github.com/OmicsML/dance/blob/v1.0.0-

benchmark/dance/transforms/spatial_feature.py), among many others

(https://github.com/OmicsML/dance/tree/v1.0.0-benchmark/dance/transforms). These rich  transformations make it easier for researchers to build their methods on top of the effective  components from previous state-of-the-art methods.

3. **Dataset Hub**: DANCE serves as a dataset hub, offering diverse "starter packs" for researchers to  explore various single-cell analysis tasks. Moreover, DANCE data objects now seamlessly  interface with AnnData and MuData objects, ensuring compatibility within the AnnData  ecosystem (see replies above). This unified interface simplifies access to vast amounts of single cell data.

In summary, DANCE provides (1) standardized model framework, (2) rich selection of advanced and  highly specialized data transformations, and (3) diverse options of built-in or custom data. We believe  that the combination of these features positions DANCE as a valuable resource that can significantly  expedite the development of novel computational methods, advancing the field of single-cell analysis.

**Reviewer’s Comment**: Minor 1. Table 1, what is the meaning of Benchmark datasets and task specific  algorithms? At least scanpy and seurat contain some datasets. Is Leiden / Louvain a task specific  algorithms? If so, scanpy & seurat should also have a mark.

**Authors’ Response:** We appreciate the reviewer’s comment regarding comparison between DANCE  and other popular single-cell libraries and toolkits. Benchmark datasets here refer to standardized  datasets that are commonly used in the field of that task for evaluation. The task specific algorithms are  algorithms specifically designed to resolve the task. I agree with you that scanpy and seurat contain some  datasets for a limited number of tasks and support a few algorithms like Leiden / Louvain. I have marked  them in the revised manuscript. Thank the reviewer for pointing this out.

**Reviewer’s Comment**: Minor 2. Why the dataset class for cell type annotation task is named as  scDeepSort datasets? It's weird.

**Authors’ Response:** We agree with the reviewer's point. We have renamed the dataset object to  CellTypeAnnotationDataset instead for clarity. Anecdotally, the cell type annotation datasets all came  from the scDeepSort paper, hence the dataset object's naming. However, we do agree that it is quite

confusing. Furthermore, we have more cell type annotation datasets beyond those compiled by  scDeepSort. Thus, we have made the corresponding change to the dataset object.

**Reviewer #2**

**Reviewer’s Comment**: Major 1. The manuscript briefly mentions that DANCE provides a variety of  ways for graph construction. The weight of each edge between each cell node and its corresponding  gene node is determined by gene counts, and there is no edge linked between any pairs of cell or gene  nodes. However, the details of constructing the weighted heterogeneous cell-to-gene graph are not  clearly explained. It will be better if they could provide more explanations for the construction of graphs.

**Authors’ Response:** We appreciate the reviewer’s comment regarding the supported graph  construction in DANCE. We have provided more details about graph construction from the graph-sc  model accordingly in the revised manuscript. Please also refer to more types of graph construction  supported in DANCE here: https://pydance.readthedocs.io/en/latest/api/transforms.graph.html.

**Reviewer’s Comment**: Major 2. In the Single modality module - Cell type annotation on page 11, the  authors mention that the performance of their SingleCellnet and ACTINN is lower than the reported  performance from the paper on Mouse Brain 2695. And the accuracy of SingleCellnet and ACTINN is also  lower in the additional file 5 Figure 1: b. What is the cause of this state of things? The authors should  also consider trying more datasets or multiple metrics to evaluate their methods.

**Authors’ Response:** We appreciate the reviewer’s comment regarding the performance difference  between our implementation and the original implementation. In most cases, the performance of our  reimplementation is similar to the original implementation. Sometimes our reimplementation is better  than the original ones, but sometimes it can be worse than the original ones as you mentioned above.

These differences may arise from language implementation (python VS R), python version, package  version, operating systems, and other reasons.

We are trying our best to make the difference as small as possible and make the performance of our  reimplementation more convincing via collecting more benchmark datasets for comprehensive  evaluation and executing more metric evaluation as we did for multimodal integration in the revision.  Taking joint embedding task as an example, we add additional Biology Conservation Metrics and Batch  Removal Metrics for comprehensive evaluation in the revised manuscript.

Biology Conservation Metrics:

● Normalized mutual information (NMI) metric: compares the overlap of two clusterings ● Cell cycle conservation score metric: serves as a proxy for the preservation of the signal  associated with gene programs during data integration.

Batch Removal Metrics:

● Batch ASW metric: is used to quantify batch mixing by taking into account the incompatibility of  batch labels per cell type cluster.

● Graph connectivity metric metric: determines whether cells of the same kind from various  batches are embedded close to one another.

Please refer to the joint embedding task in the revised manuscript for more performance comparison  under different metrics.

**Reviewer’s Comment**: Major 3. In the manuscript on page 14, lines 42-44. The authors state that  most of the models' performances on two slices are not reported individually in the original paper. This  statement is inaccurate, the performance on 12 slices is compared inside these model supplement  materials. The authors only provide a comparison of performance on the 151507, 151673, and 151676  slices, and there is no improvement in the ARI of 151673 in Additional file 5 Figure 3: a. The authors  should consider more convincing performance comparisons in all 12 slices of the DLPFC dataset.

**Authors’ Response:** We thank the reviewer for pointing this out. The performance on 12 slices is  individually compared in the supplementary. We have corrected the statement in the revised  manuscript. In addition, we have further conducted experiments on all 12 slices and reported average  performance on 12 slices from DANCE in the revised manuscript. Please refer to performance showup  section and Additional file 5 (Figure 6) in the revised manuscript. Thank the reviewer for pointing this  out.

**Reviewer’s Comment**: Major 4. Table 2 summarizes all supported benchmark datasets in DANCE.  What are the criteria for benchmarking data selection? As sequencing technology advances and more  single-cell datasets become available, how can this model be applied to new datasets?

**Authors’ Response:** We thank the reviewer for the constructive comments. All supported 21  benchmark datasets across 8 benchmark tasks in the DANCE are commonly used benchmark datasets to  evaluate each task, and they are obtained from state-of-art methods under each task. For example,  spatialLIBD dataset supported in DANCE is commonly used as a standard benchmark dataset to evaluate  cell type deconvolution in SpaGCN, STAGATE and stLearn. 10X PBMC 4K, Mouse Bladder Cells, Worm  Neuron Cells, Mouse Embryonic Stem Cells datasets are widely used to evaluate clustering in graph-sc,  scDCC, scDeepCluster and scDSC.

For the second question, how can this model be applied to new datasets?

● All of our models in DANCE are reimplemented with a unified interface. For example, data  loading is executed in a generic way via **dataloader.load_data()**, and

**model.preprocessing_pipeline()** works for all datasets and models to specify model specific  preprocessing functions. Because our DANCE package is compatible with unified anndata data  objects, end users can easily format their own task specific datasets and add them into DANCE  for further evaluation.

● We have additionally provided a short tutorial for the DANCE data object, including how to use  the built-in datasets, as well as how to construct a DANCE dataset object using user-given  AnnData (https://github.com/OmicsML/dance/blob/v1.0.0-

benchmark/notebooks/dance_data_tutorial.ipynb)

● Our DANCE team is also continually collecting additional benchmarking datasets across the 8  tasks from new studies.

● By open-sourcing DANCE, we welcome contributions from the community to bring in more  datasets. We strive to make the contribution process feel welcoming and as accessible as  possible by providing a short contribution guideline to help those who want to contribute  (Additional file 4 - Contribution Instructions in DANCE ).

**Reviewer’s Comment**: Major 5. Could DANCE support the addition of R language-based models for  benchmarking? And if so, the applicability of the models will be improved. How to perform  hyperparameter tuning experiments on the newly added model?

**Authors’ Response:** We thank the reviewer for the question. We note that all models in DANCE are  reimplemented to fit in a unified development environment based on the Python language with primary  deep learning packages including PyTorch, DGL and PyG. What’s more, all models in DANCE have generic  interfaces for usage.

If the original implementation is R language-based, our DANCE team will help re-implement them in  Python language for public usage. Python and deep learning libraries exhibit notable advantages in  facilitating GPU acceleration, particularly when dealing with a substantial quantity of single-cell data.

**Hyperparameter tuning experiments on the newly added model**: once the newly added model is added  into DANCE, end users can easily execute the command line by specifying the search space of any  interested parameter in the command line for hyperparameter tuning.

Here we take DSTG model on CARD synthetic benchmark dataset under Cell Type Deconvolution task as  an example:

The above shows all parameters of DSTG model potentially tuned to find best performance. End users  can specify a search value for each interested parameter in the command line. For example, setting the  number of neurons in the latent layer to be 16, learning rate to be 0.001 and graph node filter number  to be 50 in the DSTG model as shown below to see how well it is.

Kindly remind that the hyperparameter tuning in current DANCE is compatible with the hyperparameter  optimization framework like Optuna. So the end user can easily perform hyperparameter tuning in the  specified searching space.

**Reviewer’s Comment**: Major 6. It will be better if the authors could make their experiment data  open access.

**Authors’ Response:** We thank the reviewer for the suggestion. All experiment datasets are open  access at https://www.dropbox.com/sh/8wn1xmjk2a0qkaj/AAAs5x-n4smmknyz5uezw7rQa?dl=0. We  have added the dataset link into the **Availability of data and materials** section in the manuscript.

**Reviewer’s Comment**: Minor 1. Please ensure that all abbreviations are fully defined and explained.  Where an English term abbreviation first appears in the manuscript, the corresponding full name should  be written first. For example, Page 12 line 52 "GEX".

**Authors’ Response:** We thank the reviewer sincerely for reviewing our study. We have checked that  all abbreviations are fully defined and explained, and updated accordingly in the revised manuscript.

**Reviewer’s Comment**: Minor 2. When an abbreviation is defined and then reappears in the  manuscript, use the abbreviation, not the full name. For example, "Deep Graph Library (DGL) and  PyTorch Geometric (PyG)" appear several times in the manuscript.

**Authors’ Response:** We thank the reviewer for careful reading and for your patience, which we  greatly appreciate. We have removed full names after the initial abbreviation definition in the revised  manuscript.

**Reviewer’s Comment**: Minor 3.It is suggested that future directions and potential areas of  improvement be provided in the conclusion section to consider additional single-cell analysis tasks to  further enhance the significance and practical utility of the DANCE.

**Authors’ Response:** We really appreciate the reviewer’s suggestion. Due to the nature of extensible  features of our DANCE platform, more additional single-cell analysis tasks, models and benchmark  datasets can be easily added and supported into the DANCE platform to further enhance the significance  and practical utility of the DANCE. We have added and updated it in the conclusion section of the  revised manuscript.

**2^nd^ round**

**Reviewer 2**

The authors have addressed all my concerns.
